# Development and Validation of the Healthy Longevity Index for Personalized Healthy Aging in Primary Care: Cross-National Retrospective Analysis

**DOI:** 10.2196/80034

**Published:** 2025-11-03

**Authors:** Hsi-Yu Lai, Shu Zhang, Rei Otsuka, Shih-Tsung Huang, Hidenori Arai, Fei-Yuan Hsiao, Liang-Kung Chen

**Affiliations:** 1 Graduate Institute of Clinical Pharmacy, College of Medicine, National Taiwan University, Taipei, Taiwan; 2 Department of Epidemiology of Aging, National Center for Geriatrics and Gerontology, Obu, Japan; 3 Department of Pharmacy, National Yang Ming Chiao Tung University, Taipei, Taiwan; 4 Center for Healthy Longevity and Aging Sciences, National Yang Ming Chiao Tung University, Taipei, Taiwan; 5 Graduate Institute of Clinical Pharmacy, College of Medicine, National Taiwan University, Taipei, Taiwan; 6 Department of Pharmacy, College of Medicine, National Taiwan University Hospital, Taipei, Taiwan; 7 Center for Geriatrics and Gerontology, Taipei Veterans General Hospital, No. 201, Sec 2, Shih-Pai Road, Taipei, 11217, Taiwan, +886 2-28757830, +886 2-28757711; 8 Taipei Municipal Gan-Dau Hospital, Taipei, Taiwan

**Keywords:** healthy longevity, disability-free survival, intrinsic capacity, primary care, precision health

## Abstract

**Background:**

Measuring and promoting healthy aging at an individual level remains challenging as promoting healthy longevity requires real-time, personalized tools to assess risk and guide interventions in clinical practice.

**Objective:**

This study aimed to develop and validate a novel Healthy Longevity Index (HLI) for use in primary care settings in older adults.

**Methods:**

Using data from the Taiwan Longitudinal Study on Aging (TLSA; n=4470), we developed a nomogram-based HLI incorporating demographics, lifestyle factors, intrinsic capacity (IC) measures, and chronic conditions to predict 4-, 8-, and 12-year disability- and dementia-free survival (absence of physical disability, dementia, or mortality). The HLI was internally validated in a TLSA subset and externally validated in the Japanese National Institute for Longevity Sciences, Longitudinal Study of Aging (NILS-LSA) cohort (n=1090).

**Results:**

The 12-year HLI nomogram demonstrated robust performance, with C-statistics of 0.79 (bootstrapped 95% CI 0.78‐0.80) in the TLSA training cohort and 0.77 (bootstrapped 95% CI 0.75‐0.79) in the TLSA validation cohort. External validation in the NILS-LSA yielded a C-statistic of 0.71 (bootstrapped 95% CI 0.66‐0.76). The HLI effectively stratified participants into risk tertiles, with the highest-risk group showing only 27.8% probability of 12-year disability- and dementia-free survival compared to 87.8% in the lowest-risk group. Key predictors included age, sex, education, and, particularly, IC impairments in locomotion, visual acuity, and cognition—all assessable during routine primary care consultations.

**Conclusions:**

The HLI provides a practical tool for real-time, personalized assessment of healthy longevity risk in primary care settings. Its design enables providers to deliver person-centered care through targeted interventions and individualized prevention strategies that promote healthy aging across populations, especially in older adults.

## Introduction

The burgeoning global demographic of older adults presents complex challenges across physical, mental, and social spheres, necessitating comprehensive strategies to optimize later-life well-being, as conceptualized by the World Health Organization (WHO)’s definition of healthy aging, that is, the enhancement and preservation of functional ability over time [1]. Consequently, primary care systems worldwide face increasing pressure to address these challenges as the first point of contact for aging populations. The paramount goal and pragmatic approach is to extend health span by minimizing disability-adjusted life years (DALYs) throughout the life course [2]. While DALYs constitute a robust metric for quantifying unhealthy life years at a macro level, their derivation is contingent upon extensive public health data. Moreover, DALYs are inherently lagging indicators, typically available with a 2-year temporal offset from the reference period. Besides, DALYs reflect population-level status, limiting their applicability to individuals and clinical decision-making in primary care settings. In 2016, the WHO’s integrated care for older people (ICOPE) framework operationalized the measurement of intrinsic capacity (IC) as a composite biological construct to facilitate personalized care planning for healthy aging [3]. However, translating IC assessment into routine primary care practice remains challenging. Despite impairments in IC, overall or individual domain, being a substantial predisposing factor for disability and mortality, and predictive of diverse adverse outcomes [4-9], its correlation with DALYs and capacity for real-time estimation within the context of healthy aging remains ill-defined.

Technological advancements have facilitated the development of diverse real-time personal risk assessments, thereby revolutionizing health care delivery through enhanced precision, accuracy, and patient outcomes [10]. For instance, a smartwatch app effectively detected atrial fibrillation in a large population, offering potential for early intervention and improved outcomes [11]. Furthermore, a study of over 40,000 people with insulin-treated diabetes found that real-time continuous glycemic monitoring initiation led to a significant decrease in glycated hemoglobin (−0.40%), hypoglycemia (2.7% reduction), and emergency department visits for hypoglycemia, with no change in hyperglycemia or overall hospitalizations [12]. The presented examples unequivocally illustrate the beneficial effects of real-time, personalized health risk assessment, with the potential to significantly enhance health outcomes across a range of conditions. Similar technological advances could transform healthy aging assessment in primary care, where early detection and intervention are paramount for maintaining dignity, independence, and quality of life.

A key advantage of real-time health monitoring lies in its ability to track and potentially modify risk factors that influence health outcomes. In the context of healthy aging, several modifiable risk factors have been identified as crucial determinants of disability-free survival and cognitive health, including lifestyle behaviors (physical activity, smoking cessation, and alcohol consumption), nutritional status, management of chronic conditions (such as hypertension and diabetes), and maintenance of domains such as mobility and cognitive function [7,10,13-19]. However, current approaches to healthy aging assessment often fail to provide individuals with real-time feedback on how changes in these modifiable factors might influence their long-term outcomes, highlighting the need for dynamic, personalized assessment tools.

Unlike the readily quantifiable metrics used in specific disease management, such as diabetes and atrial fibrillation, IC and DALYs present challenges in real-time, personalized assessment and care planning within routine clinical practice. To prospectively estimate the potential burden of health conditions, disability-free survival has emerged as a promising outcome measure for specific disease states [20], treatments [21], and the broader context of healthy aging [22]. Although the exact correspondence between disability-free survival and DALYs warrants further elucidation, a rigorously formulated equation for the former is congruent with empirically determined DALYs [23]. On the other hand, while a previous study established age- and sex-specific reference centiles for IC as a potential indicator of aging trajectories, it solely captured the normative status of IC without quantifying the risk of adverse outcomes or encompassing other factors contributing to healthy longevity, not to mention the possibility of real-time measurements suitable for clinical implementation [5]. To facilitate proactive self-management and tailored interventions for healthy aging, this study sought to develop a novel Health Longevity Index (HLI)—a probabilistic estimate of disability- and dementia-free survival within a specified timeframe—and its corresponding nomogram using a nationally representative cohort sample. In addition, the study endeavored to substantiate the predictive capacity of the index through external validation using a Japanese population-based cohort.

## Methods

### Healthy longevity index (probability of disability- and dementia-free survival) and its nomogram construction cohort: Taiwan Longitudinal Study on Aging (TLSA)

Our HLI was deliberately theory-driven based on the WHO’s conceptualization of healthy aging (ie, IC) and fundamental health behavior. Based on the WHO’s “ICOPE Handbook of Guidance on person-centered assessment and pathways in primary care” [24], our HLI aims to screen for loss in a range of domains of IC and assess health and social care needs to develop a personalized care plan. To effectively identify individuals at risk of care dependency, the ICOPE pathway begins with ICOPE Step 1, which involves screening for failing tasks across 5 domains of IC: locomotion, vitality, sensory (visual and hearing loss), cognitive function, and psychological well-being.

**Figure 1.**
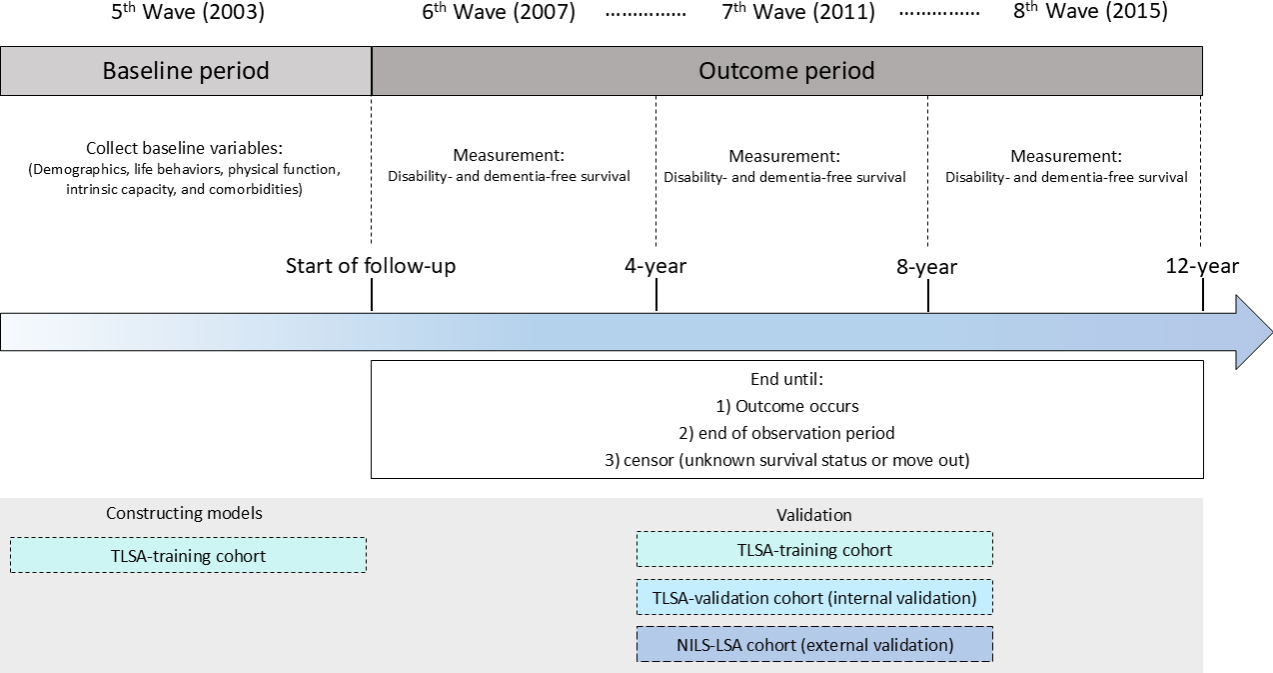
Study design of the Healthy Longevity Index (HLI). NILS-LSA: Japanese National Institute for Longevity Sciences, Longitudinal Study of Aging; TLSA: Taiwan Longitudinal Study on Aging.

The data used to develop a novel HLI (probability of disability- and dementia-free survival) and its nomogram (Figure 1) in this study were obtained from the Taiwan Longitudinal Study on Aging (TLSA), a comprehensive national survey initiated by the Health Promotion Administration of the Ministry of Health and Welfare. The TLSA was aimed at deriving deep insights into the overall well-being of middle-aged as well as older adults in Taiwan. To ensure a representative sample of the community, a 3-stage random-sampling method was systematically used, which facilitated the recruitment of participants and achieved a response rate of up to 90%. The scope of the data collection included assessments of participants’ physical activity, comorbidities, lifestyle choices, and mental health status. The details for participant recruitment and data collection have been documented in previous publications [7,10,16] and are readily available on the official Health Promotion Administration website [25]. This study was designed and reported following the TRIPOD (Transparent Reporting of a Multivariable Prediction Model for Individual Prognosis or Diagnosis) checklist.

The study cohort for constructing the nomogram consisted of 5377 participants from the fifth wave of the TLSA survey (2003). Among them, we excluded individuals residing in nursing homes (n=110), those whose responses were provided by proxies (n=328), participants with unknown residence information (n=15), with unknown BMI (n=164), and with unknown cognitive function (n=35). Given that the missingness rate was less than 5%, a complete case approach was adopted for the analysis (Figure S1 in Multimedia Appendix 1). To minimize reverse causality (ie, undiagnosed, subclinical diseases leading to disability and mortality), we further excluded participants with physical disability (n=201), cognitive disability (n=40), and those who died within the first year of follow-up (n=14). Ultimately, the study cohort included 4470 participants. For the study, this final cohort was stratified into 2 distinct groups using simple random sampling: a training set comprising 70% of the cohort (TLSA-training cohort), which was used for model construction, and a validation set (TLSA-validation cohort) constituting the remaining 30% for further internal validation.

### External Validation Cohort: Japan National Institute for Longevity Sciences, Longitudinal Study of Aging (NILS-LSA)

To generalize the applicability of the proposed prediction model, we used the National Institute for Longevity Sciences, Longitudinal Study of Aging (NILS-LSA) cohort from Japan to further validate the model derived from the TLSA cohort. The NILS-LSA is a Japanese population-based prospective cohort study that collects and analyzes variables relating to normal aging and age-related diseases. The data of this study constituted age- and sex-stratified participants recruited by random sampling from Obu and Higashiura, Aichi Prefecture, Japan. The details of the NILS-LSA were previously reported and have been well-studied in previous research [26-29]. The first-wave examination of the NILS-LSA was conducted from November 1997 to April 2000 and included 2267 participants (aged 40‐79 years). These participants were followed up every 2 years and were replaced by new, age- and sex-matched randomly recruited participants when participants (aged ≤79 years) could not attend follow-up investigations. Participants aged 40 years were also newly recruited every year.

Participants for the external validation were derived from the fourth (June 2004 to July 2006) wave of the NILS-LSA. We chose this wave because this wave covers all the data needed for the analysis and has a relatively long follow-up duration. Among 1225 participants aged 60 years and older (the Mini-Mental State Examination [MMSE] was only conducted in participants aged ≥60 years), 1172 participants had not been certified as having a disability before or at the baseline survey. We further excluded 32 participants with incomplete covariate data at baseline and 50 with a MMSE score <24. Finally, a total of 1090 participants were included in the external validation analysis.

### Feature Selection to Construct the HLI and its Nomogram

The study collected a broad spectrum of features, including demographics (including fundamental lifestyle behaviors), underlying medical conditions (self-reported chronic conditions), and, most importantly, IC, to construct the nomogram. Demographic data include age, gender, geographic region of residence, educational attainment, marital status, living condition, as well as their health behaviors (current smoker and alcohol drinker). Furthermore, we include self-reported chronic conditions, encompassing cardiovascular diseases (hypertension, heart disease, and stroke), diabetes mellitus, liver disease, and renal disease. In particular, the baseline status of IC was also assessed for each participant (Table 1).

**Table 1.** Description of variables from the Taiwan Longitudinal Study on Aging (TLSA).

Variable	Description	Data type
Demographics		
Age (years)	Respondent’s age in 2003 (50-64, 65‐74, or older than 75)	Categorical (3 levels)
Sex	Respondent’s sex (male or female)	Categorical (2 levels)
Living region	Respondent’s living region (rural or urban)	Categorical (2 levels)
Education	Respondent’s completed education (illiterate, elementary, junior or senior high school, college or above)	Categorical (4 levels)
Marital status	Respondent’s marital status in 2003 (marital, divorced or separated, or never married, or widowed)	Categorical (3 levels)
Live with spouse	Does your spouse often live with you?	Categorical (binary)
Life behaviors		
Current smoker	Do you currently smoke?	Categorical (binary)
Current alcohol drinker	Do you currently drink alcohol?	Categorical (binary)
Physical activity	Do you usually exercise?	Categorical (binary)
BMI	Respondent’s BMI in 2003 (underweight [<18.5], healthy [18.5-24], overweight [24-28], obesity [≥28])	Categorical (ordered, 4 levels)
Functional status		
Difficulty in climbing stairs	Can you walk up to the second or third floor?	Categorical (binary)
Intrinsic capacity		
Locomotion impairment	Do you have difficulty walking 200-300 meters alone without any assistance or tools?	Categorical (binary)
Visual acuity impairment	How well can you perceive objects (even with glasses or contact lenses)?	Categorical (binary)
Hearing impairment	How well can you hear sounds (even with hearing aids)?	Categorical (binary)
Vitality impairment	In the past week, have you experienced a poor appetite or lack of desire to eat?	Categorical (binary)
Psychological impairment	CES-D^a^ scale (I felt that everything I did required significant effort; I had difficulty initiating tasks)	Categorical (binary)
Cognition impairment	SPMSQ^b^ (Spatial orientation: What is the name of this place?; Temporal orientation: What is the date today?)	Categorical (binary)
Self-reported chronic conditions		
Hypertension	In the past year, have you seen a doctor for this illness?	Categorical (binary)
Diabetes mellitus	In the past year, have you seen a doctor for this illness?	Categorical (binary)
Heart disease	In the past year, have you seen a doctor for this illness?	Categorical (binary)
Stroke	In the past year, have you seen a doctor for this illness?	Categorical (binary)
Liver disease	In the past year, have you seen a doctor for this illness?	Categorical (binary)
Renal disease	In the past year, have you seen a doctor for this illness?	Categorical (binary)

a CES-D: Center for Epidemiologic Studies Depression.

b SPMSQ: Short Portable Mental Status Questionnaire.

As our study participants were community-dwelling middle-aged and older adults, the IC was constructed in accordance with the WHO’s ICOPE Step 1 (screening) framework [30], encompassing 5 domains (locomotion, sensory, vitality, psychological, and cognitive function). As the ICOPE step 1 (screening) is intended to use a feasible tool to screen those with impairment of IC and recommend them to ICOPE step 2 (assessment) for further evaluation, the screening questions are all very easy to read for community-dwelling adults. This screening approach is particularly suited for primary care settings as it requires minimal time and no specialized equipment.

In Table 1, we have specified the screening questions for each domain of IC in TLSA. Our approach closely followed the original ICOPE Step 1 definitions for most domains (Table S1 in Multimedia Appendix 2). However, due to data availability, we also adapted the operationalization of the locomotion domain through expert consensus and evidence from existing literature to ensure alignment with ICOPE screening criteria while maintaining methodological rigor. The evaluation of the locomotion domain followed the Nagi questionnaire [31]. Participants who faced challenges or were unable to cover a distance of 200‐300 meters were categorized as having limited mobility, indicating impairment in the locomotion domain [32,33]. The sensory domain was further divided into visual and hearing subareas. Visual acuity was assessed by asking participants, “How well can you perceive objects (even with glasses or contact lenses)?" Those who responded with “poor” or “very poor” were categorized as experiencing visual impairment [34,35]. Hearing capacity was evaluated through self-reported hearing impairment, with individuals reporting hearing difficulties in either ear (even with a hearing aid) being classified as having impaired hearing [34,36]. For the vitality domain, a decrease in appetite was used as an indicator of nutritional status. Participants who reported experiencing a diminished appetite in the preceding week were categorized as having an impairment in the vitality domain [33]. To assess the psychological domain, 2 questions from The Center for Epidemiologic Studies Depression (CES-D) questionnaire were used: “I felt that everything I did required significant effort,” and “I had difficulty initiating tasks.” Participants who responded with “often” or “most of the time” to either of these questions were identified as having an impairment in the psychological domain [33]. To evaluate the cognition domain, 2 questions were used from the Short Portable Mental Status Questionnaire (SPMSQ) [37] related to spatial and temporal orientation. Failing to correctly answer either of these items was indicative of an impairment in the cognition domain.

### Disability- and Dementia-Free Survival

The primary endpoint of this study was disability- and dementia-free survival, a composite outcome defined by the absence of physical disability, dementia, or mortality. We further calculated survival probability and transformed it into an HLI by multiplying by 100, yielding a 0‐100 scale that reflects the likelihood of maintaining physical and cognitive function throughout the observation period. In TLSA, each participant was monitored at each wave from the baseline year of 2003 until the occurrence of incident disability and dementia, death, emigration, loss of follow-up, or the end of the study period in 2015, whichever occurred first. Survival status was ascertained from multiple sources, including the Household Registration and Conscription Information System of the Ministry of the Interior, the Death Certificate Report System of the Ministry of Health and Welfare, and survey data collected by interviewers. To evaluate physical disability, we used the validated activities of daily living scale for each participant. This scale encompasses 6 essential tasks: bathing, dressing or undressing, eating, transferring (getting out of bed standing up or sitting in a chair), ambulating within the home, and toileting [38]. Physical disability was defined as the inability to perform, having severe difficulty in performing, or requiring assistance to complete at least 1 of these 6 basic activities of daily living tasks. Dementia was assessed using the SPMSQ [37]. At the baseline wave in 2003, the 9-item version was used, while a 10-item version was used in subsequent follow-up waves, as the question “When is your birthday?” was not collected in 2003. For participants with 6 or more years of education, a cut-off of 4 or more errors was used. For those with fewer than 6 years of education, the cut-off was 6 or more errors. These cut-off points demonstrated balanced sensitivity and specificity for detecting dementia and were highly correlated with MMSE scores [39].

In NILS-LSA, incident disability and dementia were defined as a care need of level 1 or higher, as defined by the long-term care insurance (LTCI) system used in Japan [40]. We calculated the person-years of follow-up for each participant from the first participation date until the (1) date of incident disability (LTCI certification), (2) the date of death, (3) the date of moving out of Obu and Higashiura, or (4) the end of the study period (January 31, 2022), whichever occurred first. A dataset that included information on LTCI certification, death, or emigration was obtained from Obu and Higashiura. All data were transferred from the Obu and Higashiura Governments under an agreement related to the Epidemiologic Research and Privacy Protection Guideline.

### Statistical Analysis

The descriptive analysis of demographic and clinical features was performed. For categorical variables, the chi-square test was used to determine the statistical significance, whereas the Kruskal-Wallis test was used for continuous variables to assess the difference between groups.

#### Phase 1: Features Selection, Model Development, and Nomogram Construction for HLI

In the first phase of this study, we used a univariate Cox proportional hazards regression model to identify distinct potential features for 4-, 8-, and 12-year disability- and dementia-free survival to build up the HLI. A backward elimination with a *P* value threshold of .05 was then used to determine features at each follow-up to be included in the multivariate models. The method offers the advantage of evaluating the collective predictive capacity of the features involved by initially incorporating all features into the model, systematically eliminating the least significant variables at an early stage, and ensuring only the most essential variables remain in the final model [41].

Significant features were subsequently integrated into the model that develops a nomogram for predicting the probability of disability- and dementia-free survival to build up the HLI. To be noteworthy, the ultimate features also underwent manual evaluation by clinical experts to ensure that no clinically important features were omitted during the aforementioned process.

To identify optimal survival models, we compared the semiparametric Cox proportional hazards model and a range of parametric models, including Weibull, exponential, log-logistic, and log-normal models. The selection of the most suitable model was performed using the Akaike information criterion (AIC). Models demonstrating lower AIC values were considered to have superior fit characteristics. Based on these criteria, the Weibull regression model was selected. The coefficients derived from this model were subsequently used to compute scores that were allocated to variables on the nomogram. We further calculated survival probability and then transformed it into an HLI by multiplying by 100, yielding a 0‐100 scale.

The performance of the model was assessed through the Harrell concordance index (C-index) to evaluate the discriminative capacity. The Harrell C-index is an extension of the receiver operating characteristic curve specifically adapted for survival analysis and was frequently used in the validation of survival models in the context of their time-dependent properties [42], with the index higher than 0.7 suggesting good model performance. The calibration plot was constructed using smooth restricted cubic splines with 4 knots modified by a complementary log-log transformation. This method is often used in time-to-event data structures and helps reduce the number of knots and increase the likelihood of a linear relationship between the outcome probability and the linear predictor [43]. The 95% CI for the C-index was obtained from bootstrapping with 1000 resamplings to reduce overfitting bias. In addition, the Brier score was calculated to assess the overall performance of the predictive models, with bootstrapped 95% CIs derived from 1000 resamplings. The Brier score is calculated by squaring the largest difference between a predicted probability (which lies between 0 and 1) and the actual outcome (which is either 0 or 1). As a result, the score falls within the range of 0 to 1, where a lower Brier score indicates better calibration of the predictions. In addition, we generated Kaplan-Meier plots stratified into 3 groups according to tertiles of predicted probability (Tertile 1, Tertile 2, and Tertile 3) to enhance the examination of calibration accuracy. A log-rank test was used to assess differences in outcomes among these groups.

#### Phase 2: Internal Validation in TLSA and External Validation in Japan NILS-LSA Cohort

To calculate the probability of disability- and dementia-free survival, we first extracted coefficients derived from the prediction model of the TLSA (TLSA training cohort) and further applied them in the validation set from the remaining 30% of the TLSA cohort (TLSA validation cohort). To generalize the applicability of the proposed prediction model, we further used the NILS-LSA cohort from Japan to externally validate the model derived from the TLSA cohort. The overall model performance was assessed by using the Brier score, while the calibration plot and the C-statistic were obtained to determine the model’s accuracy and discriminability, respectively. The operational definition of variables incorporated into the nomogram measured in the NILS-LSA cohort (external validation) generally corresponded to those in the TLSA cohort (Table S2 in Multimedia Appendix 2). Both the interval validation sets of the TLSA and NILS-LSA cohorts were categorized into tertiles based on the predicted probabilities (Tertile 1, Tertile 2, and Tertile 3), respectively.

### Sensitivity Analysis

To obtain a more robust and unbiased estimate of the initial model’s performance metrics, including the mean C-statistic and mean Brier score, we conducted a sensitivity analysis by using 10-fold cross-validation using the TLSA cohort across 4-, 8-, and 12-year follow-up periods. All statistical analyses were performed using SAS software (version 9.4; SAS Institute) and the R (version 4.3.3) statistical package. We considered a 2-tailed *P*≤.05 to indicate statistical significance.

### Ethical Considerations

Ethical approval was granted by the Research Ethics Committee of National Taiwan University Hospital (202204025RINA). In addition, the Committee on the Ethics of Human Research of the National Center for Geriatrics and Gerontology (NCGG), Japan, approved the study protocol (1665‐3). Written informed consent was obtained from all participants before their inclusion in the study. All potentially identifying data were encrypted to protect anonymity. Only investigators who received approval from the principal investigator of each cohort and signed a data access agreement could access the data.

## Results

### Features of Study Participants

A total of 4470 community-dwelling participants in the TLSA were included for HLI model construction, and their characteristics are summarized in Table S3 in Multimedia Appendix 2. The cohort predominantly comprised individuals aged 50‐64 years (n=2451, 54.8%), followed by those aged 65‐74 years (n=986, 22.1%), and 75 years or older (n=1033, 23.1%). The cohort comprised 53.0% (n=2367) males, with over half residing in rural areas (n=2503, 56.0%), 18.1% (n=808) reporting illiteracy, and 67.7% (n=3028) engaging in regular physical activity. IC impairments were most prevalent in the psychological domain (n=939, 21%), followed by vitality (n=593, 13.3%), cognition (n=534, 12%), locomotion (n=608, 11.4%), visual acuity (n=500, 11.2%), and hearing (n=311, 7%). Cardiometabolic conditions were common in the study sample, with hypertension affecting 29.4% (n=1314) of participants, heart disease 13.7% (n=614), diabetes mellitus 11.7% (n=524), and stroke 1.8% (n=82).

For the study, the cohort was stratified into 2 distinct groups using simple random sampling: a training set comprising 70% (n=3129) of the cohort (TLSA-training cohort), which was used for model construction, and a validation set constituting the remaining 30% (n=1341) for internal validation (TLSA-validation cohort). No significant differences were observed between the TLSA training and validation cohorts regarding baseline characteristics.

During the 12-year observation period, 1131 (36.1%) participants in the TLSA-training cohort were documented as deceased or with incident physical disability or dementia, with 556 (13.6%) participants at 4 years and 821 (26.2%) participants by the 8-year observation period. Similarly, in the TLSA-validation cohort, 517 (38.5%) participants were recorded with the same outcomes over 12 years, with 230 (14.6%) participants at 4 years and 261 (26.2%) participants by the 8-year observation period. In the full dataset, the number of participants with unknown survival status or who had moved out was 305 (6.8%), 492 (11.0%), and 679 (15.2%) at the 4-, 8-, and 12-year follow-up, respectively.

### Model Specification and Features Associated With HLI

Features selection in TLSA training cohort using both univariate and multivariate Cox hazard regression analyses at the 12-year follow-up are provided in Table S4 in Multimedia Appendix 2. After excluding nonsignificant features from the univariate analysis and applying backward elimination, the multivariate analysis was conducted. The results indicated that older adults (65‐74 years: HR 2.85, 95% CI 2.40‐3.37; older than 75 years: HR 6.32, 95% CI 5.37‐7.42), female (HR 0.74, 95% CI 0.64‐0.85), higher level of education (elementary: HR 0.83, 95% CI 0.71‐0.98; junior or senior high school: HR 0.74, 95% CI 0.60‐0.91; college and above: HR 0.54, 95% CI 0.40‐0.71), current smoker (HR 1.39, 95% CI 1.20‐1.62), current drinker (HR 0.81, 95% CI 0.70‐0.94), having difficulty in climbing stairs (HR 1.24, 95% CI 1.04‐1.47), and IC impairment (locomotion: HR 1.32, 95% CI 1.10‐1.59; visual acuity: HR 1.21, 95% CI 1.03‐1.42; cognition: HR 1.20, 95% CI 1.02‐1.42) were significantly associated with 12-year disability- and dementia-free survival probability for building up HLI. Furthermore, cardiometabolic conditions were also associated with 12-year disability- and dementia-free survival, including hypertension (HR 1.14, 95% CI 1.01‐1.29), diabetes (HR 1.54, 95% CI 1.32‐1.81), and stroke (HR 1.57, 95% CI 1.14‐2.14). Liver disease (HR 1.54, 95% CI 1.24‐1.92) and renal disease (HR 1.46, 95% CI 1.17‐1.82) were also indicative of the outcome. Details of the features selected and their association with 4-year and 8-year disability- and dementia-free survival for building up HLI are also provided in Table S5 and Table S6 in Multimedia Appendix 2.

The goodness of fit was compared between the Cox regression model and 4 types of parametric survival models, including Weibull, exponential, log-logistic, and log-normal. The Weibull model had the lowest AIC across the 4-, 8-, and 12-year observation periods, denoting a better overall fit than the other models (Table S7 in Multimedia Appendix 2; Figures S2, S3, and S4 in Multimedia Appendix 1).

### Nomogram Development and Validation

**Figure 2.**
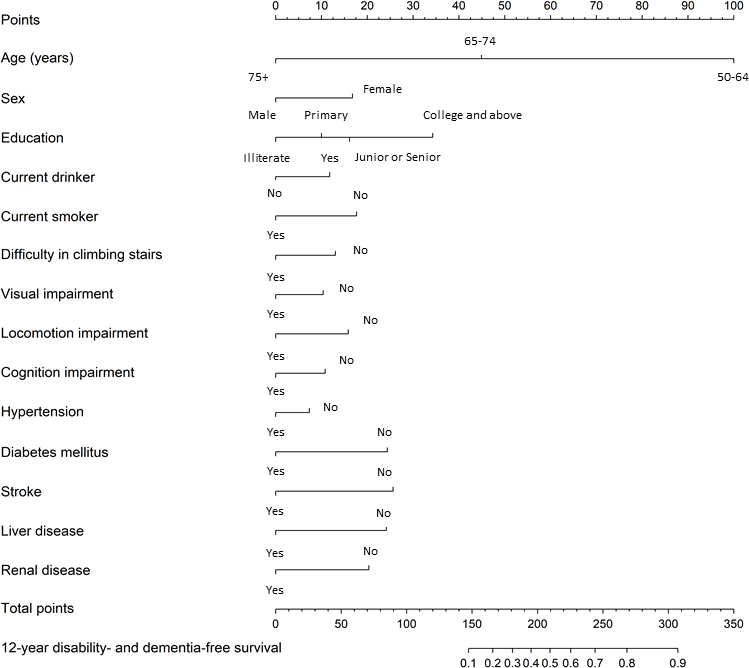
Nomogram for 12-year probability of disability- and dementia-free survival.

The features selected to construct the 12-year probability of disability- and dementia-free survival nomogram from the TLSA training cohort using the Weibull model were used to develop a nomogram. These features included demographics (age, sex, level of education, current smoking status, and current drinking status), functional status indicator (difficulty in climbing stairs), and IC (locomotion impairment, visual acuity, and cognition impairment). In addition, self-reported chronic diseases such as hypertension, diabetes mellitus, stroke, liver disease, and renal disease were incorporated (Figure 2). Nomograms for the 4-year and 8-year probabilities of disability- and dementia-free survival are presented in Figures S5 and S6 in Multimedia Appendix 1, respectively.

Consequently, the nomogram assigns points to each feature, which are used to calculate the probability of disability- and dementia-free survival, and then transformed into a HLI by multiplying by 100, yielding a 0‐100 scale. For example, a 59-year-old female with primary school education, locomotion impairment, and visual impairment would accumulate a score of 270, with scores distributed as follows: 100 points for age (50‐64 years), 17 points for being female, 10 points for having primary school education, 18 points for not smoking, 13 points for having no difficulty in climbing stairs, 11 points for having no cognitive impairment, 7 points for having no hypertension, 24 points for having no diabetes mellitus, 26 points for having no stroke, 24 points for having no liver disease, and 20 points for having no renal disease. Overall, this score predicts a 12-year HLI of 80.6. The formulas, model parameters, and the detailed weighting scores are provided in Table S8 in Multimedia Appendix 2.

The model demonstrated robust performance at the 12-year follow-up period, with consistent results observed between the TLSA training and validation cohort (Table 2). In the TLSA training cohort, the C-statistic was 0.79 (bootstrapped 95% CI 0.78‐0.80) and the Brier score was 0.16 (bootstrapped 95% CI 0.15‐0.17) at the 12-year follow-up period. Similarly, the TLSA validation cohort showed a C-statistic of 0.77 (bootstrapped 95% CI 0.75‐0.79) and a Brier score of 0.17 (bootstrapped 95% CI 0.16‐0.18), showing good discriminability. Calibration plots were used to visually assess the model’s accuracy (Figures S9 in Multimedia Appendix 1). The graphs showed good agreement in both the TLSA training and TLSA validation cohorts, indicating a well-calibrated model. In addition, the graphical presentations also confirmed good accuracy for both the TLSA training and TLSA internal validation cohorts throughout the 4-year and 8-year observation period, as provided in Figures S7 and S8 in Multimedia Appendix 1.

**Table 2.** Model performance for disability- and dementia-free survival at 4-, 8-, and 12-follow-up periods in the Taiwan Longitudinal Study on Aging (TLSA) and the National Institute for Longevity Sciences, Longitudinal Study of Aging (NILS-LSA).

Follow-up	TLSA training cohort	TLSA validation cohort	NILS-LSA validation cohort
4-year			
C-statistic (95% CI)	0.80 (0.78-0.81)	0.78 (0.74‐0.81)	0.71 (0.66-0.76)
Brier score (95% CI)	0.10 (0.09-0.11)	0.10 (0.09-0.12)	0.07 (0.06-0.08)
8-year			
C-statistic (95% CI)	0.79 (0.77-0.81)	0.77 (0.74-0.79)	0.70 (0.67-0.73)
Brier score (95% CI)	0.14 (0.13-0.15)	0.15 (0.14-0.17)	0.16 (0.15-0.17)
12-year			
C-statistic (95% CI)	0.79 (0.78-0.80)	0.77 (0.75-0.79)	0.71 (0.68-0.73)
Brier score (95% CI)	0.16 (0.15-0.17)	0.17 (0.16-0.18)	0.20 (0.19-0.21)

We further plotted Kaplan-Meier curves based on the tertiles of the 12-year predicted HLI, as generated by the nomogram. In the TLSA training cohort, the overall median predicted HLI was 74. “Tertile 3” exhibited the poorest actual survival outcomes, with a median 12-year HLI of 27.8, compared to 74.7 in “Tertile 2” and 87.8 in “Tertile 1.” Similar patterns were observed in the TLSA internal validation cohort, where the median 12-year HLI was 28.1 for “Tertile 3,” and 73.5 and 87.8 for “Tertile 2” and “Tertile 1,” respectively (Figures S10 in Multimedia Appendix 1).

### External Validation (NILS-LSA Cohort)

The models constructed were further validated in the NILS-LSA cohort from Japan using Brier score, the C-statistic, and graphical calibration plots (Figures S11 in Multimedia Appendix 1). The comparison of features included in the models between TLSA and NILS-LSA is provided in Table 3. In the NILS-LSA cohort, the statistics demonstrated good model performance with a C-statistic of 0.71 (bootstrapped 95% CI 0.66‐0.76) and a Brier score of 0.07 (bootstrapped 95% CI 0.06‐0.08) at the 4-year follow-up period. Similar results were also observed for the 8- and 12-year disability- and dementia-free survival periods (Table 2).

**Table 3.** Comparison of variables included in final models between the Taiwan Longitudinal Study on Aging (TLSA) and the National Institute for Longevity Sciences, Longitudinal Study of Aging (NILS-LSA).

Variable	TLSA training cohort (n=3129), n (%)	TLSA validation cohort (n=1341), n (%)	NILS-LSA validation cohort (n=1090), n (%)
Demographics			
Age (years)			
50‐64	1715 (54.8)	736 (54.9)	0 (0)
65‐74	681 (21.8)	305 (22.7)	756 (69.4)
75+	733 (23.4)	300 (22.4)	334 (30.6)
Sex			
Female	1475 (47.1)	628 (46.8)	554 (50.8)
Education			
Illiterate	556 (17.8)	252 (18.8)	0 (0)
Elementary	1511 (48.3)	635 (47.4)	20 (1.8)
Junior or senior	751 (24.0)	320 (23.9)	799 (73.3)
College and above	311 (9.9)	134 (10.0)	271 (24.9)
Life behaviors			
Current smoker	722 (23.1)	306 (22.8)	137 (12.6)
Current alcohol drinker	1000 (32.0)	430 (32.1)	529 (48.5)
Functional status			
Difficulty in climbing stairs	484 (15.5)	209 (15.6)	123 (11.3)
Intrinsic capacity			
Locomotion impairment	350 (11.2)	158 (11.8)	104 (9.5)
Visual acuity impairment	341 (10.9)	159 (11.9)	169 (15.5)
Vitality impairment	401 (12.8)	192 (14.3)	188 (17.2)
Psychological impairment	643 (20.6)	296 (22.1)	94 (8.6)
Cognition impairment	373 (11.9)	161 (12.0)	109 (10.0)
Self-reported chronic conditions			
Hypertension	926 (29.6)	388 (28.9)	453 (41.6)
Diabetes mellitus	370 (11.8)	154 (11.5)	111 (10.2)
Stroke	59 (1.9)	23 (1.7)	64 (5.9)
Liver disease	182 (5.8)	93 (6.9)	65 (6.0)
Renal disease	160 (5.1)	78 (5.8)	60 (5.5)

We also plotted Kaplan-Meier curves based on the tertiles of the 12-year HLI in the NILS-LSA cohort (Figure S10 in Multimedia Appendix 1). The overall median predicted 12-year HLI was 59.5. “Tertile 3” exhibited a median 12-year HLI of 32.6, compared to 59.5 in “Tertile 2” and 71.5 in “Tertile 1.”

### Developing the Real-Time, Personalized HLI Platform

**Figure 3.**
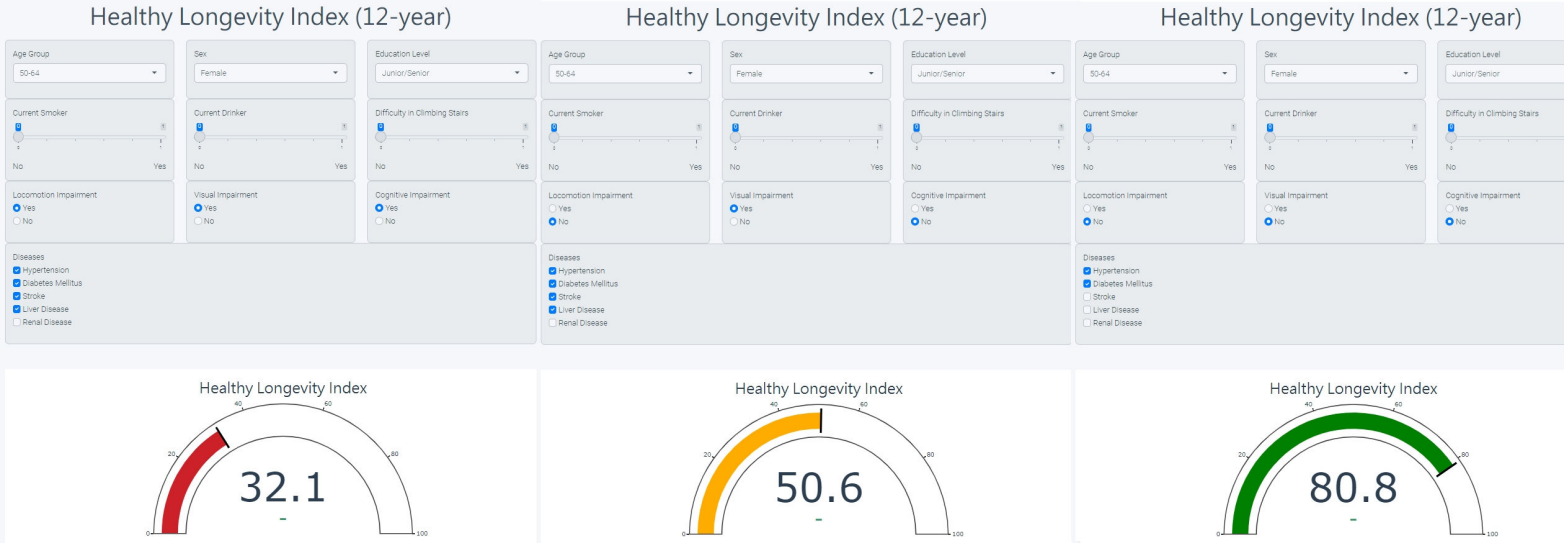
Real-time dashboard of personalized Healthy Longevity Index.

A user-friendly app was developed to optimize accessibility and facilitate real-world implementation. The real-time model is presented in a dashboard format, as provided in Figure 3. This personalized application calculates the HLI using the prediction model of specific timeframes, stratifying individuals into 3 distinct risk groups: high risk (Tertile 1, red light), medium risk (Tertile 2, yellow light), and low risk (Tertile 3, green light). The electronic interface is designed for use by clinical practitioners and researchers, facilitating decision-making within clinical settings and enabling personalized lifestyle modifications in response to fluctuating HLI levels.

### Sensitivity Analysis

We conducted 10-fold cross-validation in estimating the initial model’s performance metrics using the entire TLSA cohort. The results aligned with our previous findings across the 4-, 8-, and 12-year follow-up periods. Specifically, at the 4-year follow-up, the C-statistic was 0.79 (95% CI 0.77‐0.81), with a Brier score of 0.10 (95% CI 0.09‐0.11). At the 8-year follow-up, the C-statistic was 0.78 (95% CI 0.77‐0.79), and the Brier score was 0.15 (95% CI 0.14‐0.15). Similarly, at the 12-year follow-up, the C-statistic was 0.78 (95% CI 0.77‐0.79), while the Brier score increased to 0.16 (95% CI 0.16‐0.17).

## Discussion

### Principal Findings

This study introduces a novel, holistic HLI designed to promote healthy aging through real-time assessments, lifestyle optimization, and the development of personalized care plans. The HLI predicts the probability of maintaining active physical and cognitive function within specific timeframes. Data from 2 large-scale national longitudinal cohorts, the TLSA and the NILS-LSA in Japan, were used for HLI development and validation (internal and external). The nomogram integrates a multifaceted array of predictors, including demographic characteristics, lifestyle behaviors, IC measures, and chronic conditions, to generate individualized risk estimates suitable for primary care implementation. Notably, the model demonstrated robust predictive performance, with C-indices of 0.79 (95% CI 0.78‐0.80) in the TLSA training cohort and 0.71 (95% CI 0.66‐0.76) in the NILS-LSA cohort (external validation), indicating good discrimination across diverse populations. Furthermore, the electronic HLI dashboard generates a real-time individual HLI score. Primary care providers and their patients can periodically monitor this score to assess their potential for healthy longevity—defined as the transformed probability of disability- and dementia-free survival within specified timeframes—and to evaluate the impact of lifestyle modifications or targeted intervention plans.

### Comparison With Previous Work

Early detection and continuous monitoring through appropriate biomarkers have become fundamental to modern disease management, historically relying on periodic biomarker assessments to inform health care decision-making. Recent advancements in biomedical and digital technologies, coupled with the availability of population-scale datasets, have created the potential for real-time or continuous biomarker monitoring, promising a transformative impact on health management. While the efficacy of biosensors, wearable devices, and continuous glycemic monitoring in enhancing clinical outcomes has been well established, a comparable paradigm for the WHO’s healthy aging framework remains elusive. Despite the WHO’s intention to promote self-assessment, IC evaluation still heavily relies on health care professionals in community settings [44]. The HLI addresses this gap by providing primary care teams with a practical tool that can be integrated into routine consultations. Conversely, the HLI in this study was derived from questionnaire-based data (including IC assessment) and fundamental health behavior metrics, potentially serving as an effective marker for real-time monitoring to promote healthy aging via self-assessments. By incorporating a multitude of modifiable factors, including lifestyle behaviors and health conditions, the HLI enables individuals and health care providers to target interventions toward these modifiable factors, thereby enhancing the potential for healthy aging.

Regarding real-world implementation, growing evidence supports the feasibility and acceptance of digital health assessment tools among older adults in community settings. Studies have demonstrated that older adults are increasingly receptive to smartphone-based health apps and web-based questionnaire tools when they provide meaningful health information [45]. Furthermore, questionnaire-based screening tools similar to our HLI approach have been successfully implemented in primary care settings and community health programs, with evidence suggesting that older adults value receiving personalized health risk information when it is presented in an understandable format and coupled with guidance for improvement [46]. The integration of our HLI dashboard with existing health care workflows and community wellness programs could facilitate adoption while providing necessary support for interpreting results and developing action plans. In addition, research on health behavior change in older adults suggests that personalized risk feedback, when delivered appropriately, can serve as an effective motivator for lifestyle modifications rather than a source of distress, particularly when combined with self-efficacy building and social support [47]. However, while these studies demonstrate technical feasibility and user acceptance, further implementation research is needed to evaluate the real-world impact of tools such as the HLI on actual health outcomes, behavior change sustainability, and long-term well-being in diverse older adult populations.

Building on this demonstrated acceptance among older adults, an important clinical application of the HLI lies in its potential to guide referral decisions and care pathways as individuals’ risk profiles change over time. Our tertile-based risk stratification system could serve as objective thresholds to support primary care clinicians in making timely referrals for specialist assessment [48]. For instance, individuals transitioning from low-risk to moderate- or high-risk categories may benefit from comprehensive geriatric assessment, referral to memory clinics for cognitive evaluation, or enrollment in structured fall prevention programs [49]. Furthermore, the granular assessment of IC domains within the HLI could help primary care providers identify which specific specialists (geriatricians, neurologists, or rehabilitation specialists) would be most appropriate for referral based on the individual’s risk profile [50]. While we do not yet have a well-designed clinical protocol for HLI-based referrals, prioritizing interventions that target modifiable risk factors—such as lifestyle behaviors, physical activity, IC maintenance, and chronic disease management—should remain the primary focus for translating risk assessment into actionable clinical care. However, formal validation of HLI-based referral thresholds and their impact on clinical outcomes, health care usage, and cost-effectiveness will be essential before widespread clinical implementation [51].

The HLI may be implemented in primary health care settings and preventive screening among community-dwelling older adults, where the goal is early identification and prevention rather than management of established disease. This implementation context aligns well with our study population and addresses the growing need for proactive risk assessment in preventive care [51,52]. For clinical interpretation in primary care, physicians should use HLI scores as decision support for preventive interventions, health promotion activities, and determining appropriate monitoring intensity [53]. Integration with electronic health records in primary care settings will enable seamless incorporation into routine wellness visits and preventive care workflows [54,55]. While the current HLI is optimized for community-dwelling older adults, the methodological principles could be adapted to develop similar instruments for populations with specific diseases or conditions; however, such applications would require separate development and validation studies.

Among the modifiable risk factors encapsulated within the HLI, IC holds promise for addressing population health challenges related to aging within primary care systems. A recent systematic review and meta-analysis, encompassing 37 studies with a total of 206,693 participants, established a significant inverse relationship between IC and functional declines, as well as mortality risk, in older adults. These findings support the potential of IC as a biomarker of healthy aging; however, further research is necessary to refine its measurement and application within diverse populations and settings [56]. The utility of IC as a biomarker for healthy aging is limited by the inconclusive causal link between IC and DALYs, and the challenges associated with self-assessment and monitoring of IC. The INSPIRE Project has established age- and sex-specific reference centiles for IC using data from 975 adults aged 20‐102 years to offer a framework for individuals to assess their healthy aging trajectory, but the limitations to developing real-time personalized assessment persist. The calculation of centiles and the comparative nature of individual scores within the cohort do not provide explicit probabilities of healthy aging. Furthermore, evaluating the efficacy of intervention programs using this approach presents methodological challenges [18,57].

### Limitations and Future Directions

Despite the development and validation of the HLI as a tool for web-based, real-time, personalized assessment of healthy aging status, several limitations warrant consideration. First, the relatively healthy profiles of participants in the TLSA and NILS-LSA cohorts may have introduced a selection bias toward more favorable outcomes. However, it universally exists in all cohort studies. Second, while this study aligned with the WHO’s conceptualization of healthy aging by focusing on physical and cognitive capacity, the exclusion of social and other mental well-being dimensions necessitates further research to establish a comprehensive, consensual operational definition of healthy aging. Third, while both the cognition predictor and dementia outcome derive from the SPMSQ, we used only 2 orientation questions for the predictor versus the full SPMSQ score with education-adjusted cutoffs for the outcome. Although this represents different levels of cognitive assessment, we acknowledge this methodological approach may have some impact on results for cognitive-related outcomes. Future studies using independent cognitive assessment tools would provide stronger validation of these findings. Fourth, the integration of novel digital biomarkers, such as activity patterns, voice, and facial expression recognition, into the HLI framework would further enhance real-time risk assessment and intervention monitoring. Fifth, future studies should evaluate the HLI’s implementation in diverse primary care settings and assess its impact on clinical decision-making, patient outcomes, and health care resource usage.

### Conclusion

This study successfully developed and validated the HLI using large-scale nationally representative cohort data from Taiwan and Japan, which demonstrates potential as a real-time and personalized biomarker for healthy aging in primary care practice. By providing a more dynamic monitoring approach, the index offers greater responsiveness to interventions and lifestyle modifications in real-world settings. The integration of readily accessible digital biomarkers from wearable devices is anticipated to further refine the index’s accuracy and utility as a mobile tool for promoting healthy longevity within primary care systems.

## Supplementary material

10.2196/80034 Multimedia Appendix 1 Additional figures.

10.2196/80034 Multimedia Appendix 2 Additional tables.

## Abbreviations

AIC: Akaike information criterion
C-index: concordance index
CES-D: Center for Epidemiologic Studies Depression
DALY: disability-adjusted life year
HLI: Healthy Longevity Index
IC: intrinsic capacity
ICOPE: integrated care for older people
LTCI: long-term care insurance
MMSE: Mini-Mental State Examination
NILS-LSA: National Institute for Longevity Sciences, Longitudinal Study of Aging
SPMSQ: Short Portable Mental Status Questionnaire
TLSA: Taiwan Longitudinal Study on Aging
TRIPOD: Transparent Reporting of a Multivariable Prediction Model for Individual Prognosis or Diagnosis
WHO: World Health Organization
