# Supplementary material for: Development and Validation of the Healthy Longevity Index for Personalized Healthy Aging in Primary Care: Cross-National Retrospective Analysis
Source: JMIR Aging. 2025 Nov 3;8:e80034. doi: 10.2196/80034 (PMC12975412; doi:10.2196/80034)

Development and Validation of Healthy Longevity Index for personalized healthy ageing in Primary Care: A Cross-Nation Retrospective analysis

Hsi-Yu Lai, MS[1,2], Shu Zhang, PhD[3], Rei Otsuka, PhD[3], Shih-Tsung Huang, PhD [4,5], Hidenori Arai, MD, PhD[6], Fei-Yuan Hsiao, PhD [1,7,8], Liang-Kung Chen, MD, PhD [5,9,10]

[1]Graduate Institute of Clinical Pharmacy, College of Medicine, National Taiwan University, Taipei, Taiwan; [2]School of Pharmacy, College of Medicine, National Taiwan University, Taipei, Taiwan; [3]Department of Epidemiology of Aging, Center for Gerontology and Social Science, National Center for Geriatrics and Gerontology, Obu, Japan; [4] Department of Pharmacy, National Yang Ming Chiao Tung University, Taipei, Taiwan; [5]Center for Healthy Longevity and Aging Sciences, National Yang Ming Chiao Tung University, Taipei, Taiwan;[6]National Center for Geriatrics and Gerontology, Obu, Japan; [7]School of Pharmacy, College of Medicine, National Taiwan University, Taipei, Taiwan; [8]Department of Pharmacy, National Taiwan University Hospital, Taipei, Taiwan; [9]Center for Geriatrics and Gerontology, Taipei Veterans General Hospital, Taipei, Taiwan; [10]Taipei Municipal Gan-Dau Hospital (Managed by Taipei Veterans General Hospital);

Table of Contents

Figure S1. Participant flowchart. 2

Figure S2. Hazard ratios (HRs) and 95% CIs of the association of factors with disability, dementia, and mortality over 4 years in the training cohort assessed by the Weibull model. 3

Figure S3. Hazard ratios (HRs) and 95% CIs of the association of factors with disability, dementia, and mortality over 8 years in the TLSA-training cohort assessed by the Weibull model. 4

Figure S4. Hazard ratios (HRs) and 95% CIs of the association of factors with disability, dementia, and mortality over 12 years in the TLSA-training cohort assessed by the Weibull model. 5

Figure S5. Nomogram for 4-year probability of disability- and dementia-free survival. 6

Figure S6. Nomogram for 8-year probability of disability- and dementia-free survival. 7

Figure S7. Calibration plots for Weibull models (A) 4-year predicted risk of disability, dementia, and mortality for TLSA-training cohort (B) 4-year predicted risk of disability, dementia, and mortality for TLSA-validation cohort. 8

Figure S8. Calibration plots for Weibull models (A) 8-year predicted risk of disability, dementia, and mortality for TLSA-training cohort (B) 8-year predicted risk of disability, dementia, and mortality for TLSA-validation cohort. 9

Figure S9. Calibration plots for Weibull models (A) 12-year predicted risk of disability, dementia, and mortality for TLSA-training cohort (B) 12-year predicted risk of disability, dementia, and mortality for TLSA-validation cohort. 10

Figure S10. Kaplan-Meier curves plots stratified by tertile group (A) 12-year disability- and dementia-free survival for TLSA-training cohort (B) 12-year disability- and dementia-free survival for TLSA-validation cohort (C) 12-year disability- and dementia-free survival for NILS-LSA cohort. 11

Figure S11. Calibration plots for Weibull models predicted the risk of death or disability for the NILS-LSA cohort. (A) 4-year predicted risk of disability, dementia, or mortality. (B) 8-year predicted risk of disability, dementia, or mortality. (C) 12-year predicted risk of disability, dementia, or mortality. 12

Figure S1. Participant flowchart.

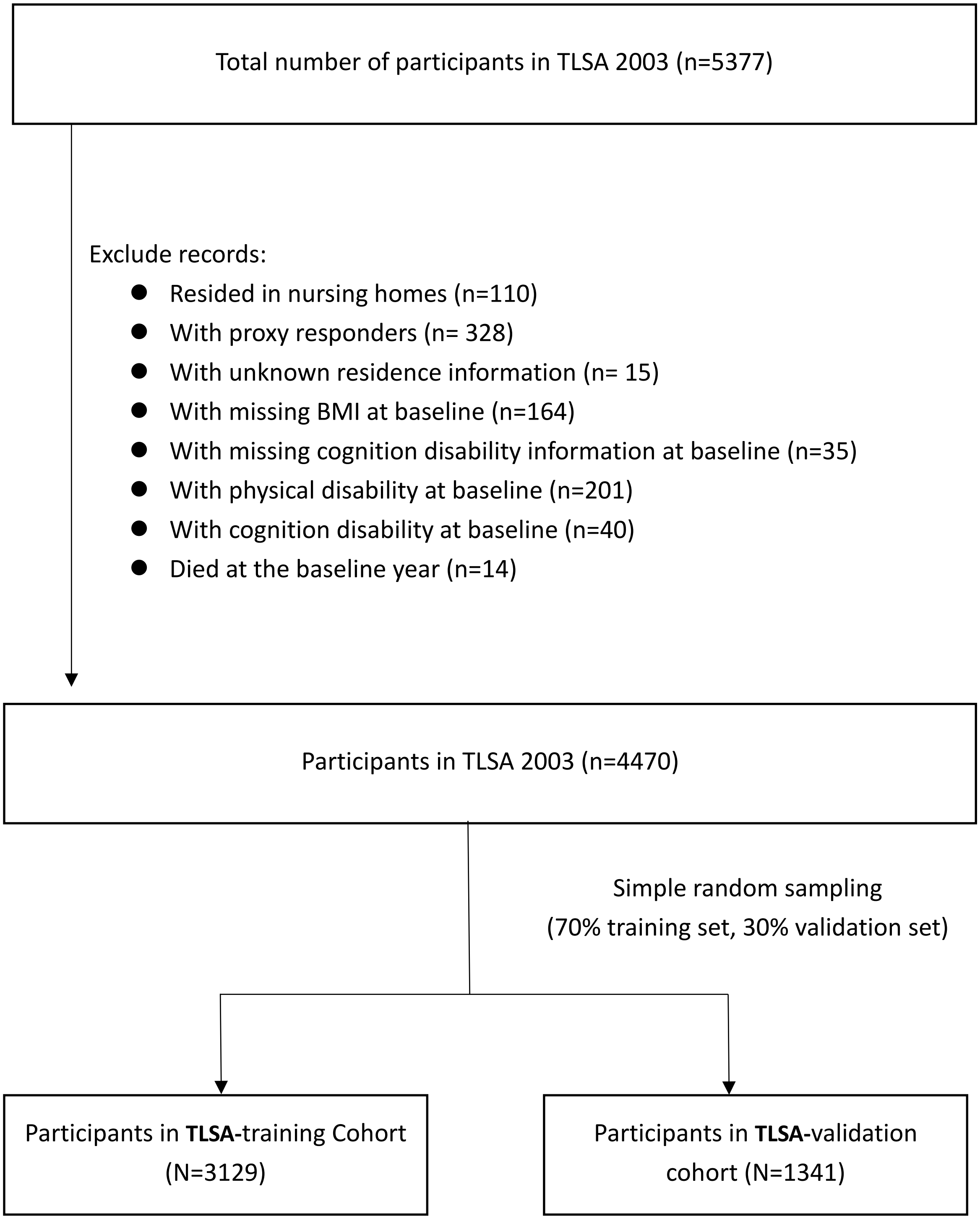

Figure S2. Hazard ratios (HRs) and 95% CIs of the association of factors with disability, dementia, and mortality over 4 years in the training cohort assessed by the Weibull model.

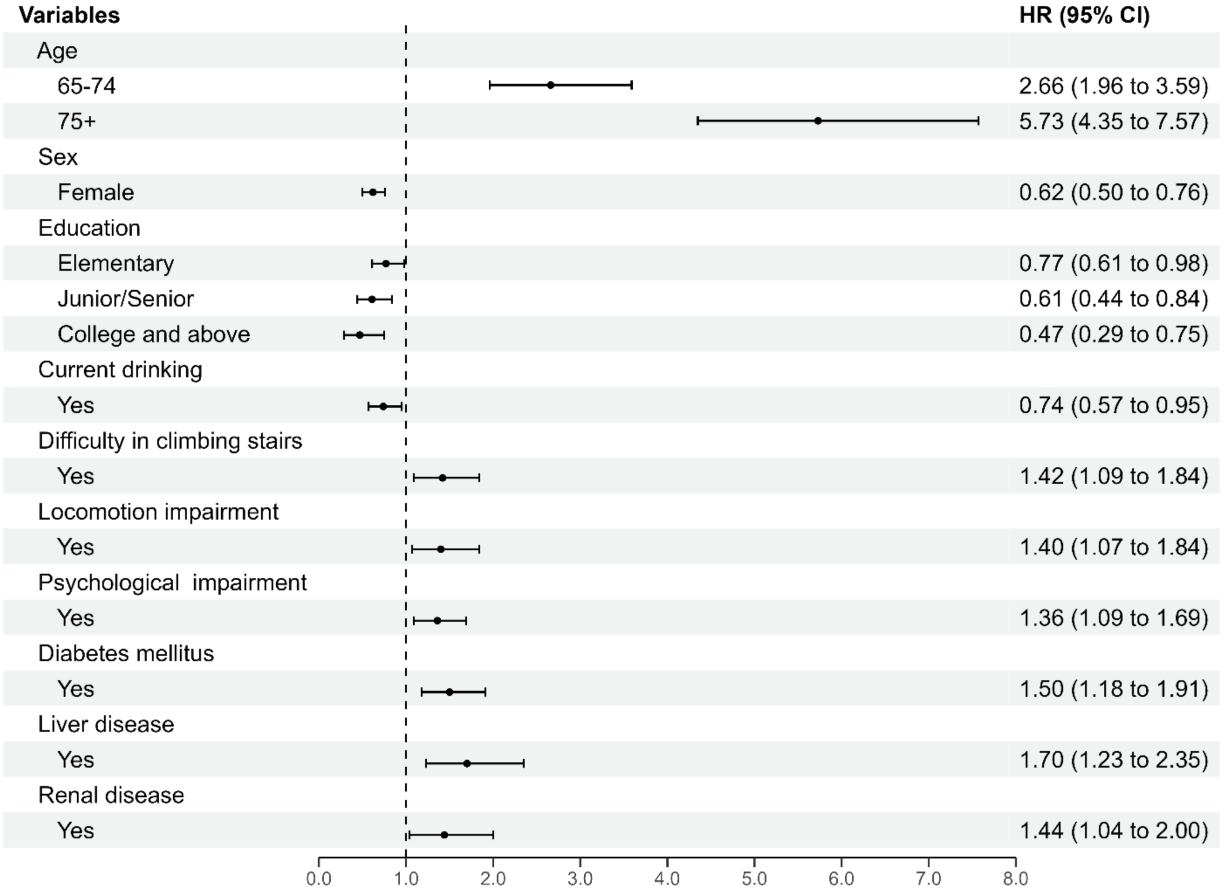

Figure S3. Hazard ratios (HRs) and 95% CIs of the association of factors with disability, dementia, and mortality over 8 years in the TLSA-training cohort assessed by the Weibull model.

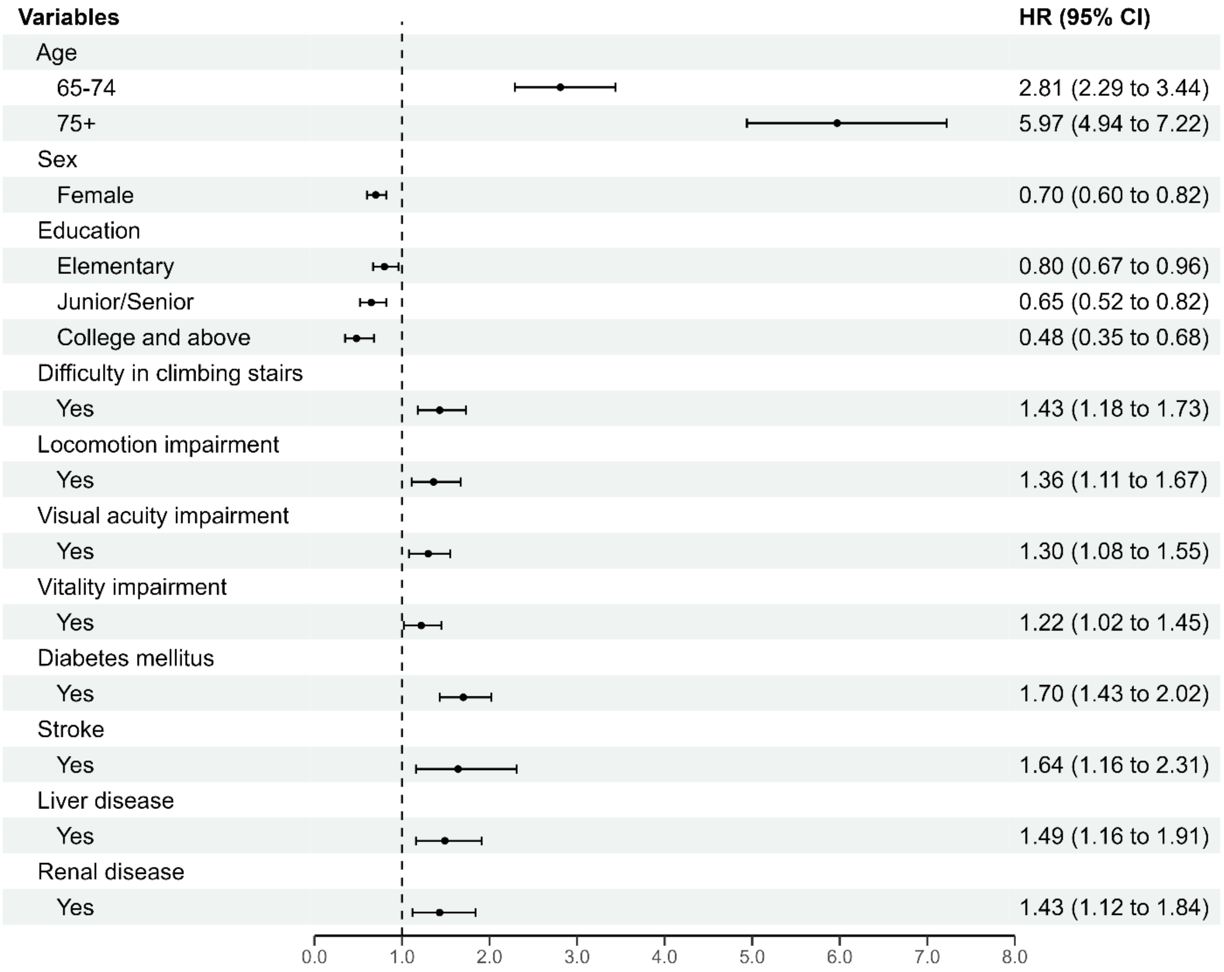

Figure S4. Hazard ratios (HRs) and 95% CIs of the association of factors with disability, dementia, and mortality over 12 years in the TLSA-training cohort assessed by the Weibull model.

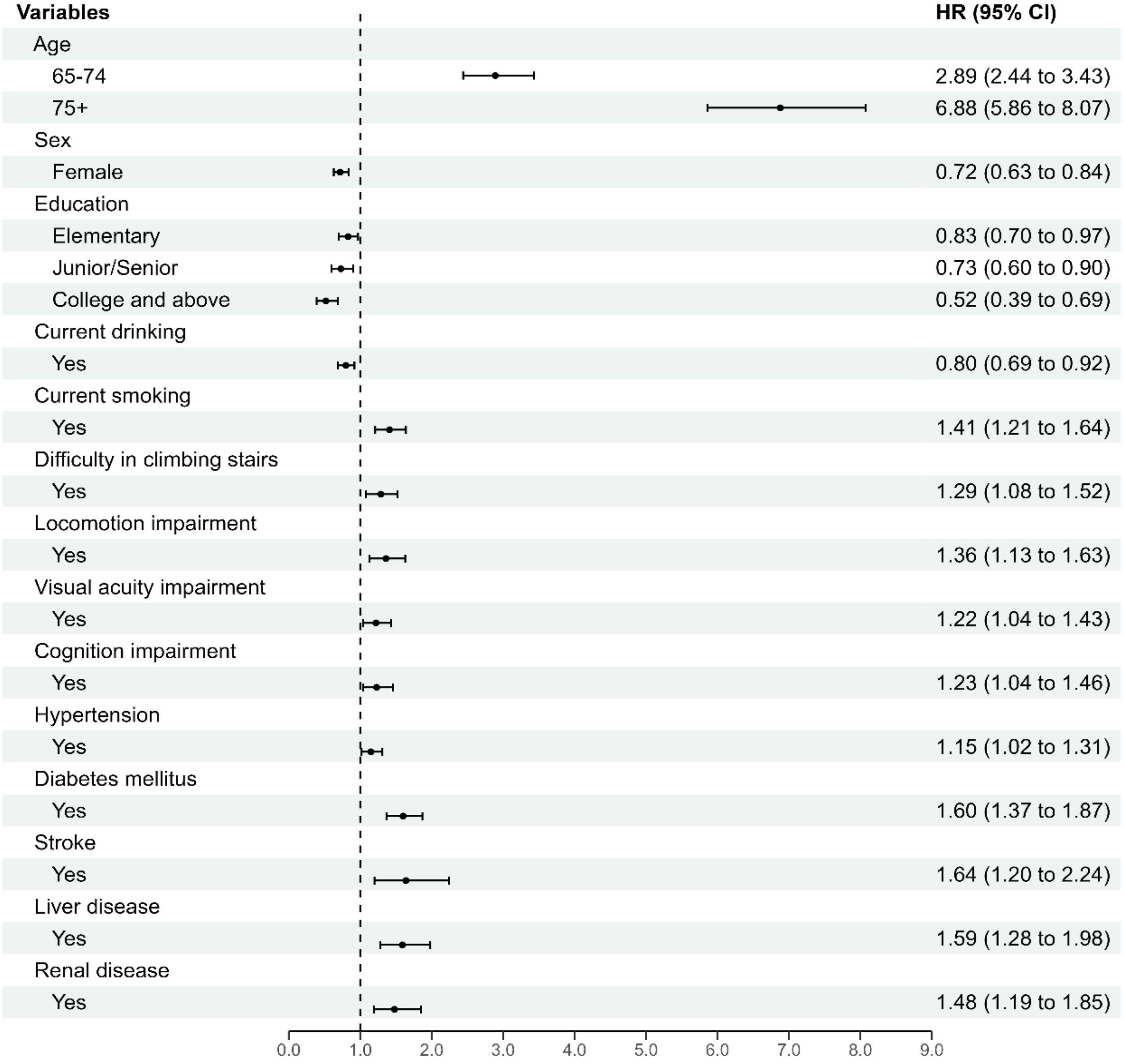

Figure S5. Nomogram for 4-year probability of disability- and dementia-free survival.

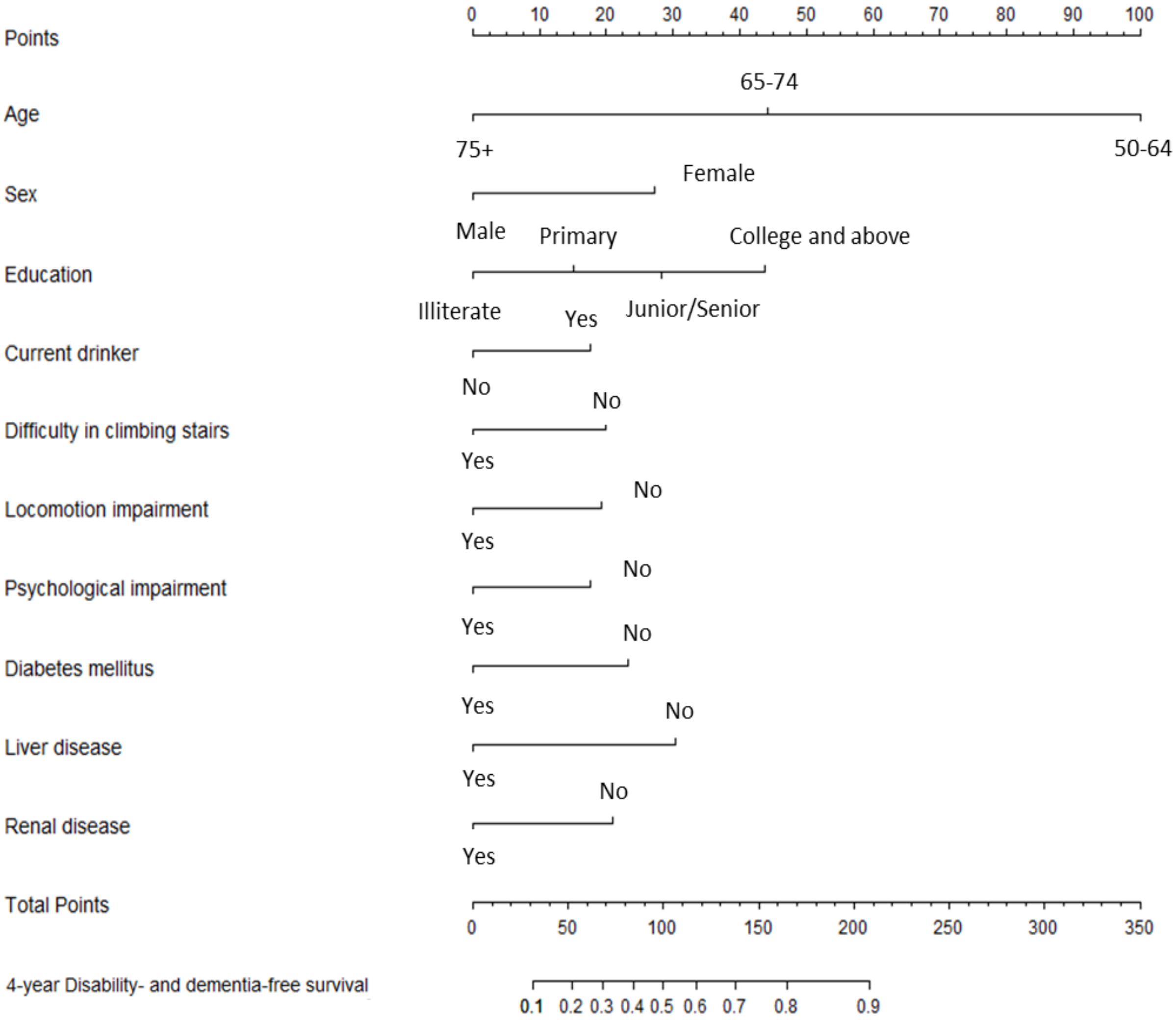

Figure S6. Nomogram for 8-year probability of disability- and dementia-free survival.

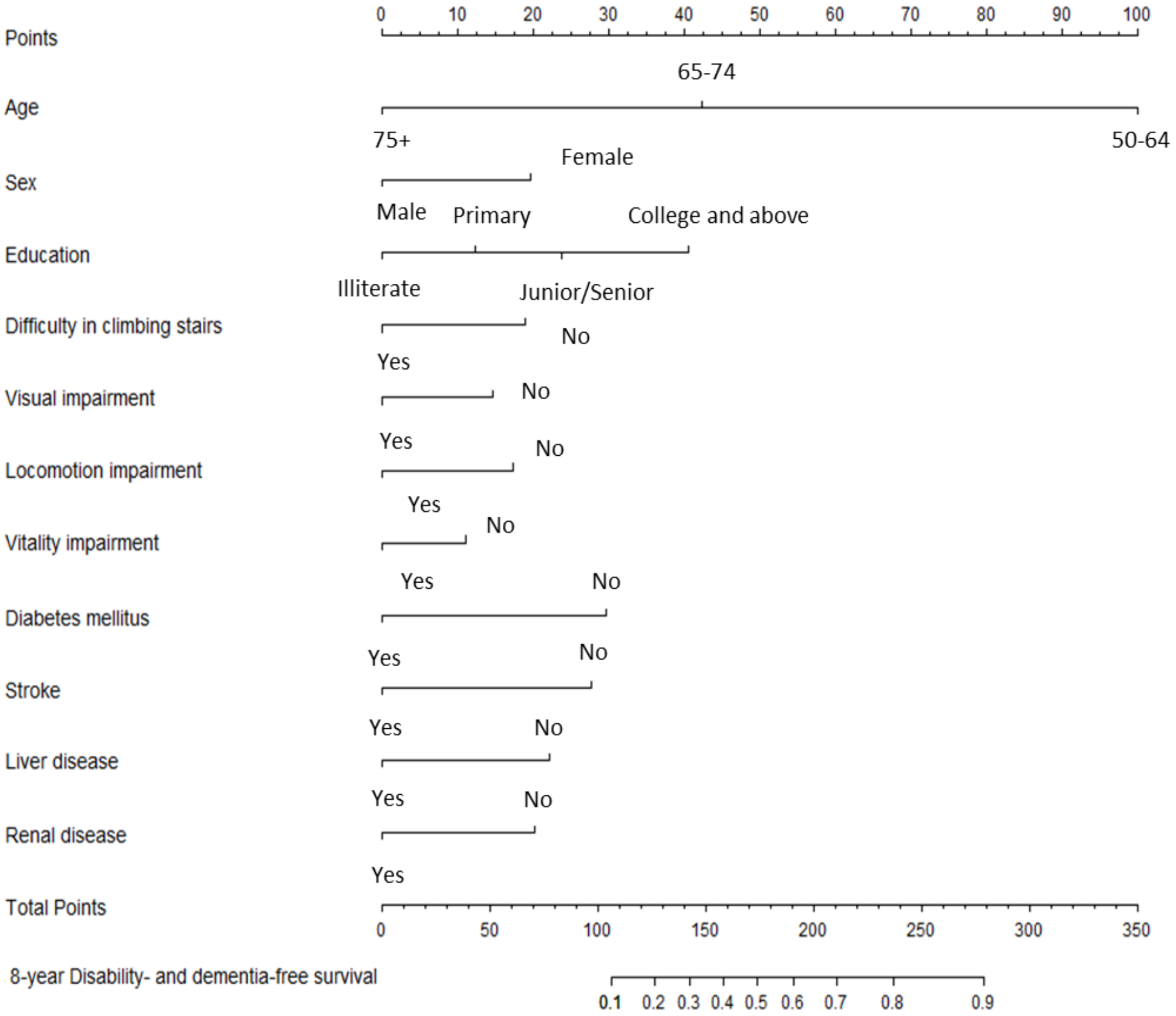

Figure S7. Calibration plots for Weibull models (A) 4-year predicted risk of disability, dementia, and mortality for TLSA-training cohort (B) 4-year predicted risk of disability, dementia, and mortality for TLSA-validation cohort.

(A)

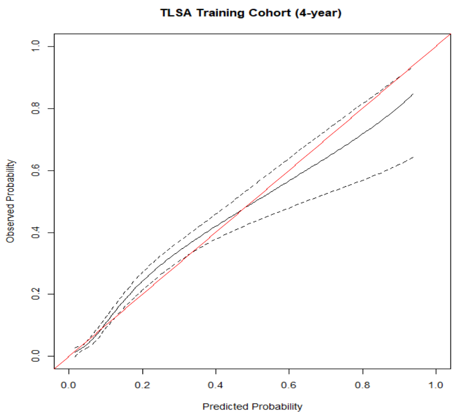

(B)

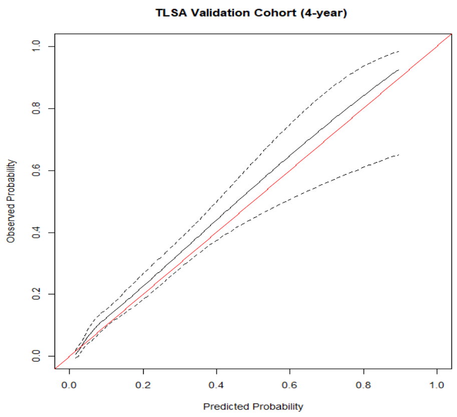

Figure S8. Calibration plots for Weibull models (A) 8-year predicted risk of disability, dementia, and mortality for TLSA-training cohort (B) 8-year predicted risk of disability, dementia, and mortality for TLSA-validation cohort.

(A)

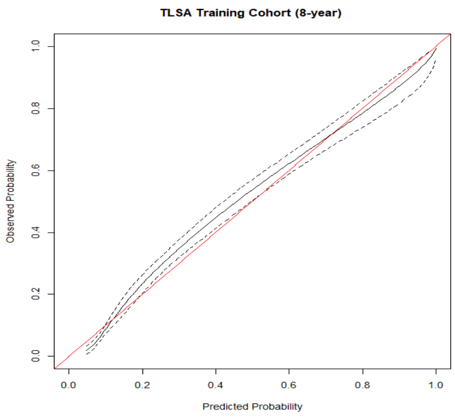

(B)

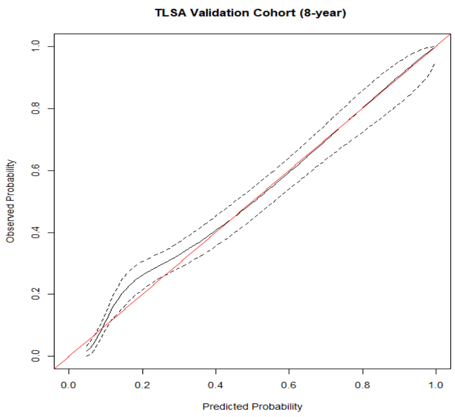

Figure S9. Calibration plots for Weibull models (A) 12-year predicted risk of disability, dementia, and mortality for TLSA-training cohort (B) 12-year predicted risk of disability, dementia, and mortality for TLSA-validation cohort.

(A)

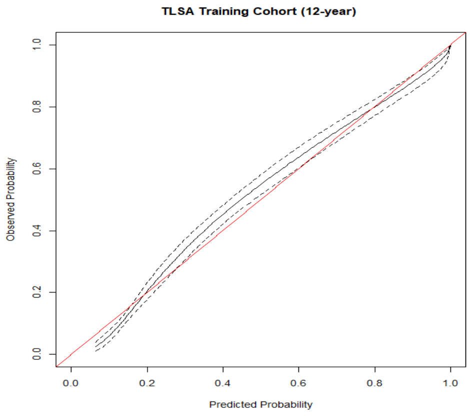

(B)

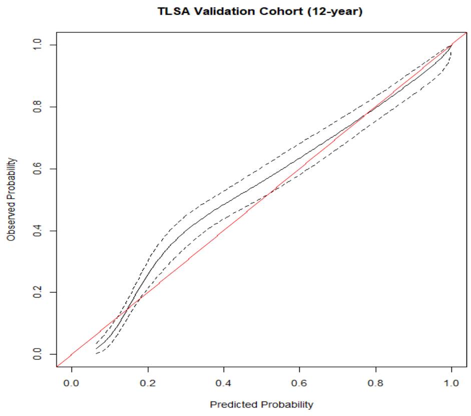

Figure S10. Kaplan-Meier curves plots stratified by tertile group (A) 12-year disability- and dementia-free survival for TLSA-training cohort (B) 12-year disability- and dementia-free survival for TLSA-validation cohort (C) 12-year disability- and dementia-free survival for NILS-LSA cohort.

(A)

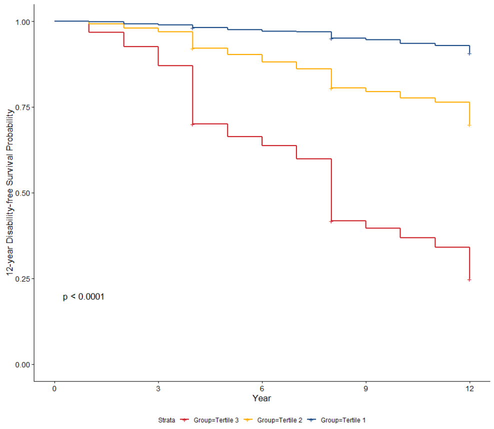

(B)

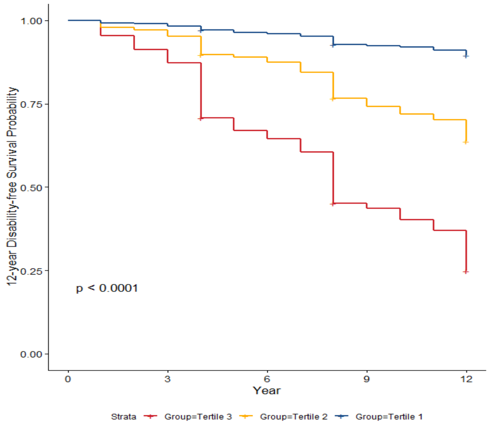

(C)

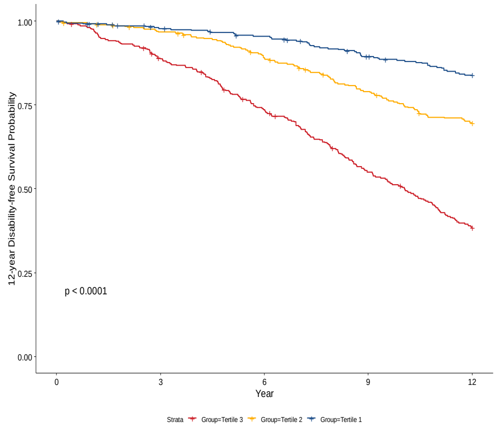

Figure S11. Calibration plots for Weibull models predicted the risk of death or disability for the NILS-LSA cohort. (A) 4-year predicted risk of disability, dementia, or mortality. (B) 8-year predicted risk of disability, dementia, or mortality. (C) 12-year predicted risk of disability, dementia, or mortality.

(A)

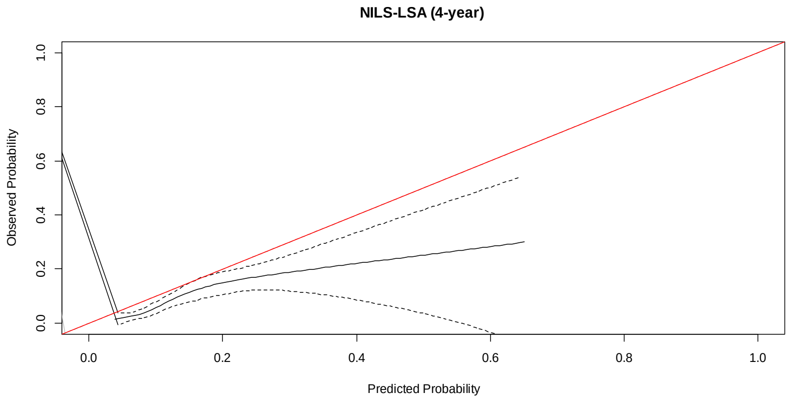

(B)

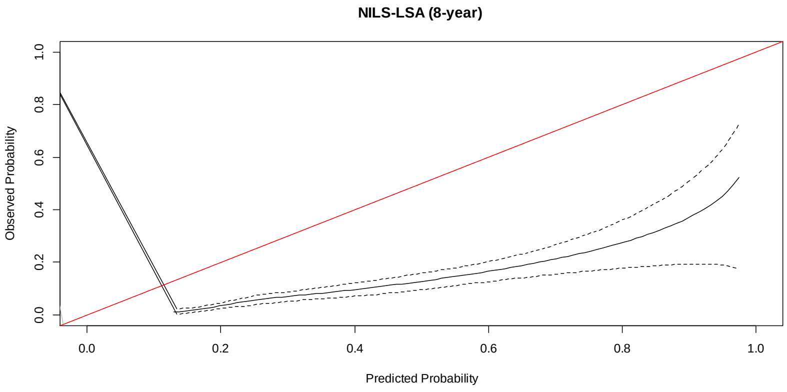

(C)

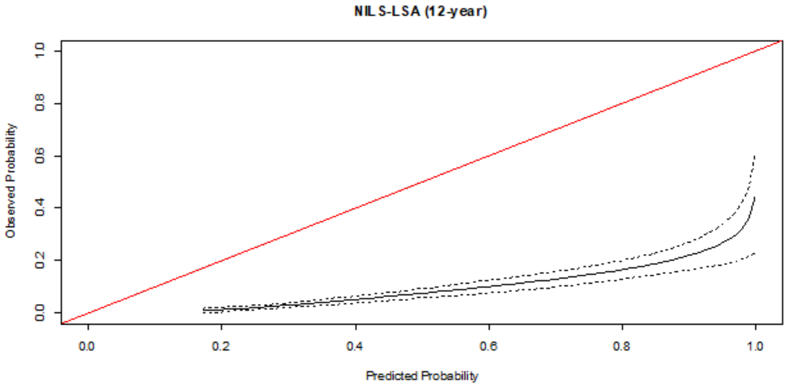

Supplement: Multimedia Appendix 1 [file aging-v8-e80034-s001.pdf]
