# Supplementary material for: Development and Validation of the Healthy Longevity Index for Personalized Healthy Aging in Primary Care: Cross-National Retrospective Analysis
Source: JMIR Aging. 2025 Nov 3;8:e80034. doi: 10.2196/80034 (PMC12975412; doi:10.2196/80034)
Supplement: Multimedia Appendix 2 [file aging-v8-e80034-s002.pdf]

Development and Validation of Healthy Longevity Index for personalized healthy ageing in Primary Care: A Cross-Nation Retrospective analysis

Hsi-Yu Lai, MS[1,2], Shu Zhang, PhD[3], Rei Otsuka, PhD[3], Shih-Tsung Huang, PhD [4,5], Hidenori Arai, MD, PhD[6], Fei-Yuan Hsiao, PhD [1,7,8], Liang-Kung Chen, MD, PhD [5,9,10]

[1]Graduate Institute of Clinical Pharmacy, College of Medicine, National Taiwan University, Taipei, Taiwan; [2]School of Pharmacy, College of Medicine, National Taiwan University, Taipei, Taiwan; [3]Department of Epidemiology of Aging, Center for Gerontology and Social Science, National Center for Geriatrics and Gerontology, Obu, Japan; [4] Department of Pharmacy, National Yang Ming Chiao Tung University, Taipei, Taiwan; [5]Center for Healthy Longevity and Aging Sciences, National Yang Ming Chiao Tung University, Taipei, Taiwan;[6]National Center for Geriatrics and Gerontology, Obu, Japan; [7]School of Pharmacy, College of Medicine, National Taiwan University, Taipei, Taiwan; [8]Department of Pharmacy, National Taiwan University Hospital, Taipei, Taiwan; [9]Center for Geriatrics and Gerontology, Taipei Veterans General Hospital, Taipei, Taiwan; [10]Taipei Municipal Gan-Dau Hospital (Managed by Taipei Veterans General Hospital);

Table of Contents

Table S1. The operational definition of intrinsic capacity included in models in TLSA and NILS-LSA compared with ICOPE step 1 (screening) 3

Table S2. Operational definition of variables included in models in NILS-LSA cohort 4

Table S3. Baseline characteristics of TLSA (training and validation cohort) 6

Table S4. Features selection for 12-year disability- and dementia-free survival by Cox regression model; TLSA (training cohort) 11

Table S5. Features selection for 4-year disability- and dementia-free survival by Cox regression model; TLSA (training cohort) 17

Table S6. Features selection for 8-year disability- and dementia-free survival by Cox regression model; TLSA (training cohort) 23

Table S7. Comparison of model fits in TLSA training cohort 29

Table S8. Model parameters, weighting score and formulas of 4-,8-,12-follow-up period in TLSA training cohort. 31

Table S1. The operational definition of intrinsic capacity included in models in TLSA and NILS-LSA compared with ICOPE step 1 (screening).

Domain	WHO's ICOPE Step 1	TLSA	NILS-LSA
**Locomotion**	Chair rise test: Did the person complete five chair rises within 14 seconds?	Do you have difficulty walking 200 to 300 meters alone without any assistance or tools	Do you have difficulty walking hundreds of meters?
**Sensory (visual impairment)**	Do you have any problems with your eyes: difficulties in seeing far, reading, eye diseases or currently under medical treatment (e.g. diabetes, high blood pressure)?	How well can you perceive objects (even with glasses or contact lenses)?	How well do you rate your visual acuity (even with glasses or contact lenses)?
**Vitality (malnutrition)**	Appetite loss: Have you experienced a loss of appetite?	In the past week, have you experienced a poor appetite or a lack of desire to eat?	In the past week, I have experienced a poor appetite or a lack of desire to eat?
**Psychological**	Over the past two weeks, have you been bothered by a) feeling down, depressed or hopeless?	I felt that everything I did required significant effort	I felt that everything I did required significant effort
	Little interest or pleasure in doing things?	I had difficulty initiating tasks	I had difficulty initiating tasks
**Cognition**	Mini mental state examination (MMSE)	SPMSQ<br>Spatial orientation:<br>What is the name of this place?<br>Temporal orientation: What's the date today?	MMSE<br>Spatial orientation: What hospital is this?<br>Temporal orientation: What's the date today (year/month/day)?

Table S2. Operational definition of variables included in models in NILS-LSA cohort.

Variable	Description
**Demographics**	
Age	Respondent's age at wave 4 (1: 60−<75, 2: ≥75)
Sex	Respondent's sex (1: male, 2: female)
Education	Education years (1: ≤6, 2: 7−12, 3: ≥13)
**Life behaviors**	
Current smoker	Do you currently smoke? (1: yes, 2: no)
Current alcohol drinker	Do you currently drink? (1: yes, 2: no)
**Function**	
Difficulty in climbing stairs	Do you have difficulty climbing stairs to the first floor?<br><br>(1: "a little bit difficult" or "very difficult", 0: "not at all")
**Intrinsic capacity**	
Locomotion impairment	Do you have difficulty walking hundreds of meters?<br><br>(1: "a little bit difficult" or "very difficult", 0: "not at all")
Visual acuity impairment	How well do you rate your visual acuity (even with glasses or contact lenses)?<br><br>(1: "poor" or "very poor", 0: "usual" or "good" or "excellent")
Vitality impairment	In the past week, I have experienced a poor appetite or a lack of desire to eat.<br><br>(1: "sometimes" or "often" or "most of the time", 0: "almost none")

Psychological impairment	Center for Epidemiologic Studies Depression scale<br>-I felt that everything I did required significant effort<br>-I had difficulty initiating tasks<br>(1: "often" or "most of the time" for any of the questions, 0: other cases)
Cognition impairment	Mini-Mental State Examination<br>Spatial orientation: What hospital is this?<br>Temporal orientation: What's the date today (year/month/day)?<br>(1: failed to answer any of these 4 items correctly, 0: answered all 4 items correctly)
**Self-reported chronic conditions**	
Hypertension	Have you ever had this disease? (1: yes, 2: no)
Diabetes mellitus	Have you ever had this disease? (1: yes, 2: no)
Stroke	Have you ever had this disease? (1: yes, 2: no)
Liver disease	Have you ever had this disease? (1: yes, 2: no)
Renal disease	Have you ever had this disease? (1: yes, 2: no)

Table S3. Baseline characteristics of TLSA (training and validation cohort).

Variable		Overall N=4470	TLSA- Training cohort N=3129	TLSA- Validation cohort N=1341	P-Value
Demographics					
Age					0.65
	50-64	2451 (54.83%)	1715 (54.81%)	736 (54.88%)	
	65-74	986 (22.06%)	681 (21.76%)	305 (22.74%)	
	75+	1033 (23.11%)	733 (23.43%)	300 (22.37%)	
Gender	Female	2103 (47.05%)	1475 (47.14%)	628 (46.83%)	0.85
Living region	Urban	1967 (44.00%)	1385 (44.26%)	582 (43.40%)	0.59
Education					0.87

Variable		Overall<br>N=4470	TLSA-<br>Training cohort<br>N=3129	TLSA-<br>Validation cohort<br>N=1341	P-Value
	Illiterate	808 (18.08%)	556 (17.77%)	252 (18.79%)	
	Elementary	2146 (48.01%)	1511 (48.29%)	635 (47.35%)	
	Junior/Senior	1071 (23.96%)	751 (24.00%)	320 (23.86%)	
	College and above	445 (9.96%)	311 (9.94%)	134 (9.99%)	
Marital status					0.36
	Marital	3323 (74.34%)	2334 (74.59%)	989 (73.75%)	
	Divorced/separated/never married	259 (5.79%)	188 (6.01%)	71 (5.29%)	
	Widowed	888 (19.87%)	607 (19.40%)	281 (20.95%)	
Live with spouse	Yes	1955 (43.74%)	1376 (43.98%)	579 (43.18%)	0.62

Variable		Overall<br>N=4470	TLSA-<br>Training cohort<br>N=3129	TLSA-<br>Validation cohort<br>N=1341	P-Value
Life behaviors					
Current smoker	Yes	1028 (23.00%)	722 (23.07%)	306 (22.82%)	0.85
Current alcohol smoker	Yes	1430 (31.99%)	1000 (31.96%)	430 (32.07%)	0.94
Physical activity	Yes	3028 (67.74%)	2109 (67.40%)	919 (68.53%)	0.46
BMI					0.56
	Underweight (<18.5)	162 (3.62%)	107 (3.42%)	55 (4.10%)	
	Healthy (18.5 to 24)	2184 (48.86%)	1529 (48.87%)	655 (48.84%)	
	Overweight (24 to 28)	1569 (35.10%)	1111 (35.51%)	458 (34.15%)	
	Obesity (> =28)	555 (12.42%)	382 (12.21%)	173 (12.90%)	

Variable		Overall N=4470	TLSA- Training cohort N=3129	TLSA- Validation cohort N=1341	P-Value
Function					
Difficulty in climbing stairs	Yes	693 (15.50%)	484 (15.47%)	209 (15.59%)	0.92
Intrinsic capacity					
Locomotion impairment	Yes	508 (11.36%)	350 (11.19%)	158 (11.78%)	0.57
Visual acuity impairment	Yes	500 (11.19%)	341 (10.90%)	159 (11.86%)	0.35
Hearing impairment	Yes	311 (6.96%)	207 (6.62%)	104 (7.76%)	0.17
Vitality impairment	Yes	593 (13.27%)	401 (12.82%)	192 (14.32%)	0.18
Psychological impairment	Yes	939 (21.01%)	643 (20.55%)	296 (22.07%)	0.25
Cognition impairment	Yes	534 (11.95%)	373 (11.92%)	161 (12.01%)	0.94

Variable		Overall N=4470	TLSA- Training cohort N=3129	TLSA- Validation cohort N=1341	P-Value
Self-reported chronic conditions					
Hypertension	Yes	1314 (29.40%)	926 (29.59%)	388 (28.93%)	0.66
Diabetes mellitus	Yes	524 (11.72%)	370 (11.82%)	154 (11.48%)	0.75
Heart disease	Yes	614 (13.74%)	413 (13.20%)	201 (14.99%)	0.11
Stroke	Yes	82 (1.83%)	59 (1.89%)	23 (1.72%)	0.70
Liver disease	Yes	275 (6.15%)	182 (5.82%)	93 (6.94%)	0.15
Renal disease	Yes	238 (5.32%)	160 (5.11%)	78 (5.82%)	0.34

Table S4. Features selection for 12-year disability- and dementia-free survival by Cox regression model; TLSA (training cohort).

	Disability- and dementia-free survival			
	Univariate		Multivariate	
	HR (95% CI)	p-value	HR (95% CI)	p-value
**Demographics**				
**Age**				
**50-64**	Ref		Ref	
**65-74**	3.55 (3.01-4.18)	<.001	2.85 (2.40-3.37)	<.001
**75+**	8.34 (7.19-9.67)	<.001	6.32 (5.37-7.42)	<.001
**Sex**				
**Male**	Ref		Ref	
**Female**	0.75 (0.66-0.84)	<.001	0.74 (0.64-0.85)	<.001
**Region**				
**Rural**	Ref			
**Urban**	0.80 (0.71-0.90)	0.003		
**Education**				
**Illiterate**	Ref		Ref	

	Disability- and dementia-free survival			
	Univariate		Multivariate	
	HR (95% CI)	p-value	HR (95% CI)	p-value
**Elementary**	0.54 (0.47-0.62)	<.001	0.83 (0.71-0.98)	0.027
**Junior/Senior**	0.43 (0.36-0.51)	<.001	0.74 (0.60-0.91)	0.003
**College and above**	0.30 (0.23-0.39)	<.001	0.54 (0.40-0.71)	<.001
**Marital status**				
**Marital**	Ref			
**Divorced/separated/never married**	1.15 (0.89-1.47)	0.29		
**Widowed**	2.07 (1.82-2.36)	<.001		
**Living status**				
**Alone**	Ref			
**With spouse**	1.65 (1.47-1.85)	<.001		
**Life behaviors**				
**Current smoker**				
**No**	Ref		Ref	
**Yes**	1.19 (1.04-1.36)	0.01	1.39 (1.20-1.62)	<.001
**Current drinking**				

	Disability- and dementia-free survival			
	Univariate		Multivariate	
	HR (95% CI)	p-value	HR (95% CI)	p-value
**No**	Ref		Ref	
**Yes**	0.66 (0.58-0.76)	<.001	0.81 (0.70-0.94)	0.004
**Physical activity**				
**No**	Ref			
**Yes**	1.18 (1.04-1.34)	0.01		
**BMI**				
**Underweight**	Ref			
**Healthy**	0.55 (0.42-0.71)	<.001		
**Overweight**	0.47 (0.36-0.62)	<.001		
**Obesity**	0.46 (0.34-0.62)	<.001		
**Function**				
**Difficulty in climbing stairs**				
**No**	Ref		Ref	
**Yes**	2.78 (2.44-3.17)	<.001	1.24 (1.04-1.47)	0.01
**Intrinsic capacity**				

		Disability- and dementia-free survival			
		Univariate		Multivariate	
		HR (95% CI)	p-value	HR (95% CI)	p-value
**Locomotion impairment**					
	**No**	Ref		Ref	
	**Yes**	2.88 (2.50-3.33)	<.001	1.32 (1.10-1.59)	0.003
**Visual acuity impairment**					
	**No**	Ref		Ref	
	**Yes**	2.12 (1.82-2.47)	<.001	1.21 (1.03-1.42)	0.02
**Hearing impairment**					
	**No**	Ref			
	**Yes**	2.56 (2.13-3.07)	<.001		
**Vitality impairment**					
	**No**	Ref			
	**Yes**	1.82 (1.56-2.11)	<.001		
**Psychological impairment**					
	**No**	Ref			
	**Yes**	1.35 (1.18-1.55)	<.001		

	Disability- and dementia-free survival			
	Univariate		Multivariate	
	HR (95% CI)	p-value	HR (95% CI)	p-value
**Cognition impairment**				
**No**	Ref		Ref	
**Yes**	2.14 (1.84-2.48)	<.001	1.20 (1.02-1.42)	0.03
**Self-reported chronic conditions**				
**Hypertension**				
**No**	Ref		Ref	
**Yes**	1.82 (1.62-2.05)	<.001	1.14 (1.01-1.29)	0.004
**Diabetes mellitus**				
**No**	Ref		Ref	
**Yes**	1.97 (1.69-2.30)	<.001	1.54 (1.32-1.81)	<.001
**Heart disease**				
**No**	Ref			
**Yes**	1.95 (1.69-2.25)	<.001		
**Stroke**				
**No**	Ref		Ref	

	Disability- and dementia-free survival			
	Univariate		Multivariate	
	HR (95% CI)	p-value	HR (95% CI)	p-value
**Yes**	3.06 (2.26-4.15)	0.001	1.57 (1.14-2.14)	0.05
**Liver disease**				
**No**	Ref		Ref	
**Yes**	1.65 (1.33-2.05)	<.001	1.54 (1.24-1.92)	<.001
**Renal disease**				
**No**	Ref		Ref	
**Yes**	1.85 (1.49-2.30)	0.001	1.46 (1.17-1.82)	<.001

Table S5. Features selection for 4-year disability- and dementia-free survival by Cox regression model; TLSA (training cohort).

	Disability- and dementia-free survival			
	Univariate		Multivariate	
	HR (95% CI)	p-value	HR (95% CI)	p-value
**Demographics**				
**Age**				
**50-64**	Ref		Ref	
**65-74**	3.52 (2.62-4.74)	<.001	2.65 (1.96-3.59)	<.001
**75+**	8.30 (6.42-10.74)	<.001	5.69 (4.31-7.51)	<.001
**Sex**				
**Male**	Ref		Ref	
**Female**	0.79 (0.65-0.95)	0.01	0.63 (0.50-0.78)	<.001
**Living region**				
**Rural**	Ref			
**Urban**	0.82 (0.68-1.00)	0.05		
**Education**				
**Illiterate**	Ref		Ref	

	Disability- and dementia-free survival			
	Univariate		Multivariate	
	HR (95% CI)	p-value	HR (95% CI)	p-value
**Elementary**	0.51 (0.41-0.63)	<.001	0.77 (0.61-0.98)	0.03
**Junior/Senior**	0.36 (0.27-0.48)	<.001	0.62 (0.45-0.85)	<.001
**College and above**	0.27 (0.17-0.43)	<.001	0.47 (0.29-0.76)	<.001
**Marital status**				
**Marital**	Ref			
**Divorced/separated/never married**	1.13 (0.74-1.71)	0.58		
**Widowed**	2.04 (1.66-2.51)	<.001		
**Living status**				
**Alone**	Ref			
**With spouse**	1.45 (1.20-1.76)	<.001		
**Life behaviors**				
**Current smoker**				
**No**	Ref			
**Yes**	1.11 (0.89-1.38)	0.38		
**Current drinking**				

	Disability- and dementia-free survival			
	Univariate		Multivariate	
	HR (95% CI)	p-value	HR (95% CI)	p-value
**No**	Ref		Ref	
**Yes**	0.53 (0.41-0.67)	<.001	0.73 (0.57-0.94)	0.02
**Physical activity**				
**No**	Ref			
**Yes**	1.14 (0.93-1.40)	0.22		
**BMI**				
**Underweight**	Ref			
**Healthy**	0.64 (0.42-0.98)	0.04		
**Overweight**	0.57 (0.37-0.89)	0.01		
**Obesity**	0.43 (0.26-0.72)	0.01		
**Function**				
**Difficulty in climbing stairs**				
**No**	Ref		Ref	
**Yes**	3.45 (2.84-4.20)	<.001	1.41 (1.09-1.83)	0.01
**Intrinsic capacity**				

		Disability- and dementia-free survival			
		Univariate		Multivariate	
		HR (95% CI)	p-value	HR (95% CI)	p-value
**Locomotion impairment**					
	**No**	Ref		Ref	
	**Yes**	3.44 (2.79-4.24)	<.001	1.40 (1.06-1.83)	0.02
**Visual acuity impairment**					
	**No**	Ref			
	**Yes**	2.34 (1.85-2.95)	<.001		
**Hearing impairment**					
	**No**	Ref			
	**Yes**	2.70 (2.07-3.52)	<.001		
**Vitality impairment**					
	**No**	Ref			
	**Yes**	2.22 (1.78-2.78)	<.001		
**Psychological impairment**					
	**No**	Ref		Ref	
	**Yes**	1.75 (1.43-2.15)	<.001	1.35 (1.09-1.68)	0.006

	Disability- and dementia-free survival			
	Univariate		Multivariate	
	HR (95% CI)	p-value	HR (95% CI)	p-value
**Cognition impairment**				
**No**	Ref			
**Yes**	2.42 (1.93-3.02)	<.001		
**Self-reported chronic conditions**				
**Hypertension**				
**No**	Ref			
**Yes**	1.72 (1.42-2.09)	<.001		
**Diabetes mellitus**				
**No**	Ref		Ref	
**Yes**	2.08 (1.65-2.63)	<.001	1.50 (1.18-1.90)	<.001
**Heart disease**				
**No**	Ref			
**Yes**	1.83 (1.45-2.31)	<.001		
**Stroke**				
**No**	Ref			

	Disability- and dementia-free survival			
	Univariate		Multivariate	
	HR (95% CI)	p-value	HR (95% CI)	p-value
**Yes**	3.22 (2.12-4.91)	<.001		
**Liver disease**				
**No**	Ref		Ref	
**Yes**	1.89 (1.38-2.60)	<0.001	1.68 (1.22-2.33)	0.002
**Renal disease**				
**No**	Ref		Ref	
**Yes**	2.14 (1.55-2.94)	<.001	1.44 (1.04-1.99)	0.03

Table S6. Features selection for 8-year disability- and dementia-free survival by Cox regression model; TLSA (training cohort).

	Disability- and dementia-free survival			
	Univariate		Multivariate	
	HR (95% CI)	p-value	HR (95% CI)	p-value
**Demographics**				
**Age**				
**50-64**	Ref		Ref	
**65-74**	3.54 (2.90-4.33)	<.001	2.80 (2.29-3.44)	<.001
**75+**	7.95 (6.66-9.49)	<.001	5.74 (4.75-6.93)	<.001
**Sex**				
**Male**	Ref		Ref	
**Female**	0.79 (0.69-0.91)	<.001	0.71 (0.61-0.83)	<.001
**Rural**	Ref			
**Urban**	0.79 (0.69-0.91)	0.01		
**Education**				
**Illiterate**	Ref		Ref	
**Elementary**	0.53 (0.45-0.63)	<.001	0.82 (0.69-0.97)	0.02

	Disability- and dementia-free survival			
	Univariate		Multivariate	
	HR (95% CI)	p-value	HR (95% CI)	p-value
**Junior/Senior**	0.40 (0.33-0.49)	<.001	0.67 (0.53-0.84)	<.001
**College and above**	0.30 (0.22-0.40)	<.001	0.50 (0.36-0.70)	<.001
**Marital status**				
**Marital**	Ref			
**Divorced/separated/never married**	1.14 (0.85-1.54)	0.38		
**Widowed**	2.04 (1.75-2.37)	<.001		
**Living status**				
**Alone**	Ref			
**With spouse**	1.57 (1.37-1.80)	<.001		
**Life behaviors**				
**Current smoker**				
**No**	Ref			
**Yes**	1.11 (0.94-1.30)	0.21		
**Current drinking**				
**No**	Ref			

	Disability- and dementia-free survival			
	Univariate		Multivariate	
	HR (95% CI)	p-value	HR (95% CI)	p-value
**Yes**	0.65 (0.55-0.76)	<.001		
**Physical activity**				
**No**	Ref			
**Yes**	1.12 (0.97-1.30)	0.13		
**BMI**				
**Underweight**	Ref			
**Healthy**	0.52 (0.38-0.70)	<.001		
**Overweight**	0.46 (0.34-0.63)	<.001		
**Obesity**	0.45 (0.32-0.64)	<.001		
**Function**				
**Difficulty in climbing stairs**				
**No**	Ref		Ref	
**Yes**	3.16 (2.73-3.66)	<.001	1.37 (1.13-1.67)	0.001
**Intrinsic capacity**				
**Locomotion impairment**				

	Disability- and dementia-free survival			
	**Univariate**		**Multivariate**	
	HR (95% CI)	p-value	HR (95% CI)	p-value
**No**	Ref		Ref	
**Yes**	3.17 (2.70-3.72)	<.001	1.32 (1.08-1.63)	0.008
**Visual acuity impairment**				
**No**	Ref		Ref	
**Yes**	2.29 (1.93-2.72)	<.001	1.29 (1.08-1.55)	0.005
**Hearing impairment**				
**No**	Ref			
**Yes**	2.49 (2.02-3.07)	<.001		
**Vitality impairment**				
**No**	Ref		Ref	
**Yes**	2.03 (1.72-2.41)	<.001	1.22 (1.02-1.46)	0.03
**Psychological impairment**				
**No**	Ref			
**Yes**	1.50 (1.29-1.75)	<.001		
**Cognition impairment**				

	Disability- and dementia-free survival			
	Univariate		Multivariate	
	HR (95% CI)	p-value	HR (95% CI)	p-value
**No**	Ref			
**Yes**	2.12 (1.79-2.52)	<.001		
**Self-reported chronic conditions**				
**Hypertension**				
**No**	Ref			
**Yes**	1.84 (1.60-2.11)	<.001		
**Diabetes mellitus**				
**No**	Ref		Ref	
**Yes**	2.14 (1.81-2.54)	<.001	1.67 (1.41-1.99)	<.001
**Heart disease**				
**No**	Ref			
**Yes**	2.03 (1.72-2.40)	<.001		
**Stroke**				
**No**	Ref		Ref	
**Yes**	3.04 (2.17-4.27)	<.001	1.58 (1.12-2.23)	0.009

		Disability- and dementia-free survival			
		Univariate		Multivariate	
		HR (95% CI)	p-value	HR (95% CI)	p-value
**Liver disease**					
	**No**	Ref		Ref	
	**Yes**	1.67 (1.31-2.14)	<.001	1.46 (1.14-1.88)	0.003
**Renal disease**					
	**No**	Ref		Ref	
	**Yes**	1.98 (1.55-2.53)	<.001	1.42 (1.10-1.82)	0.006

Table S7. Comparison of model fits in TLSA training cohort.

	AIC	BIC
**4-year**		
Cox proportional hazard	6326.26	6378.94
Weibull	2747.07	2837.80
Exponential	3307.12	3397.84
Log-normal	2956.04	3046.77
Log-logistic	2829.12	2919.85
**8-year**		
Cox proportional hazard	12066.81	12132.76
Weibull	5881.62	5978.40
Exponential	6351.37	6448.14
Log-normal	5988.57	6085.35
Log-logistic	5944.64	6041.42
**12-year**		
Cox proportional hazard	16453.27	16538.79
Weibull	8272.35	8387.27
Exponential	8719.61	8834.53

Log-normal	8356.95	8471.87
Log-logistic	8572.12	8687.04

AIC= akaike information criterion; BIC= bayesian information criterion

Table S8. Model parameters, weighting score and formulas of 4-,8-,12-follow-up period in TLSA training cohort.

	4-year		8-year		12-year	
	Value (SE)	Scores	Value (SE)	Scores	Value (SE)	Scores
**Age**						
50-64	Ref	100	Ref	100	Ref	100
65-74	-0.28 (0.046)	44	-0.47 (0.050)	42	-0.57 (0.049)	45
75+	-0.50 (0.047)	0	-0.81 (0.050)	0	-1.04 (0.049)	0
**Sex**						
Male	Ref	0	Ref	0	Ref	0
Female	0.14 (0.03)	27	0.16 (0.036)	20	0.17 (0.04)	17
**Education**						
Illiterate	Ref	0	Ref	0	Ref	0
Elementary	0.075 (0.035)	15	0.10 (0.04)	12	0.10 (0.045)	10
Junior/Senior	0.14 (0.047)	28	0.19 (0.05)	24	0.17 (0.056)	16
College and above	0.22 (0.070)	44	0.33 (0.078)	41	0.36 (0.079)	34
**Current drinker**						
No	Ref	0	-	-	Ref	0
Yes	0.088 (0.037)	18	-	-	0.12 (0.04)	12
**Current smoker**						

No	-	-	-	-	Ref	18
Yes	-	-	-	-	-0.18 (0.042)	0
**Difficulty in climbing stairs**						
No	Ref	20	Ref	15	Ref	13
Yes	-0.010 (0.039)	0	-0.15 (0.045)	0	-0.14 (0.047)	0
**Locomotion impairment**						
No	Ref	19	Ref	17	Ref	16
Yes	-0.096 (0.04)	0	-0.14 (0.048)	0	-0.16 (0.05)	0
**Visual acuity impairment**						
No	-	-	Ref	15	Ref	10
Yes	-	-	-0.12 (0.042)	0	-0.11 (0.04)	0
**Vitality impairment**						
No	-	-	Ref	11	-	-
Yes	-	-	-0.089 (0.041)	0	-	-
**Psychological impairment**						
No	Ref	18	-	-	-	-
Yes	-0.088 (0.03)	0	-	-	-	-
**Cognition impairment**						
No	-	-	-	-	Ref	11
Yes	-	-	-	-	-0.11 (0.047)	0
**Hypertension**						

	No	-	-	-	-	Ref	7
	Yes	-	-	-	-	-0.077 (0.035)	0
**Diabetes mellitus**							
	No	Ref	23	Ref	30	Ref	24
	Yes	-0.12 (0.035)	0	-0.24 (0.041)	0	-0.25 (0.044)	0
**Stroke**							
	No	-	-	Ref	28	Ref	26
	Yes	-	-	-0.23 (0.08)	0	-0.27 (0.086)	0
**Liver disease**							
	No	Ref	30	Ref	22	Ref	24
	Yes	-0.15 (0.048)	0	-0.18 (0.059)	0	-0.25 (0.06)	0
**Renal disease**							
	No	Ref	21	Ref	20	Ref	20
	Yes	-0.10 (0.048)	0	-0.16 (0.058)	0	-0.21 (0.061)	0

SE=standard error

The Weibull model

$$S(t) = e^{-(e^{-\beta \cdot X_i} \cdot t)^{\frac{1}{\sigma}}}$$

S(t) represents the survival probability, where β denotes the vector of coefficients, and t signifies time. The parameter σ serves as the scale parameter, while 1/σ characterizes the shape parameter

	**Components of formula**
**4-year**	$-\beta^{\cdot}X_i$ =-2.14-(*age=65-74, YES=1*)*(-0.28)- (*age>=75, YES=1*)*(-0.50)- (*female=1*)*0.14-(*elementary school, YES=1*)*0.075-(*Junior/Senior, YES=1*)*0.14-(*College and above, YES=1*)*0.22-(*current drinker, YES=1*)*0.088- (*difficulty in climbing stairs, YES=1*) *(-0.010)- (*locomotion impairment, YES=1*)*(-0.096)- (*psychological impairment, YES=1*)*(-0.088)- (*diabetes, YES=1*)*(-0.12)- (*liver disease, YES=1*)*(-0.15)- (*renal disease, YES=1*)*(-0.10)<br>σ=0.29; t=4
**8-year**	$-\beta^{\cdot}X_i$ =-2.96-(*age=65-74, YES=1*)*(-0.47)- (*age>=75, YES=1*)*(-0.81)- (*female=1*)*0.16-(*elementary school, YES=1*)*0.10-(*Junior/Senior, YES=1*)*0.19-(*College and above, YES=1*)*0.33- (*difficulty in climbing stairs, YES=1*) *(-0.15)- (*locomotion impairment, YES=1*)*(-0.14)- (*visual impairment, YES=1*)*(-0.12)- (*vitality impairment, YES=1*)*(-0.089)- (*diabetes, YES=1*)*(-0.24)- (*stroke, YES=1*)*(-0.23)- (*liver disease, YES=1*)*(-0.18)- (*renal disease, YES=1*)*(-0.16)<br>σ=0.46; t=8
**12-year**	$-\beta^{\cdot}X_i$=-3.31-(*age=65-74,YES=1*)*(-0.57)-(*age>=75, YES=1*)*(-1.04)- (*female=1*)*0.17-(*elementary school, YES=1*)*0.10-(*Junior/Senior, YES=1*)*0.17-(*College and above, YES=1*)*0.36-(*current drinker, YES=1*)*0.12-(*current smoker, YES=1*)*(-0.18)- (*difficulty in climbing stairs, YES=1*) *(-0.14)- (*locomotion impairment, YES=1*)*(-0.16)- (*visual impairment, YES=1*)*(-0.11)- (*cognition impairment, YES=1*)*(-0.11)- (*hypertension, YES=1*) *(-0.076)- (*diabetes, YES=1*)*(-0.25)- (*stroke, YES=1*)*(-0.27)- (*liver disease, YES=1*)*(-0.25)- (*renal disease, YES=1*)*(-0.21)<br>σ=0.54; t=12
**Examples**	A 59-year-old female with primary school degree, locomotion impairment, and vision impairment<br>$-\beta^{\cdot}Xi$=-3.31-0*(-0.57)-0*(-1.04)-1*0.17-1*0.10-0*0.17-0*0.36-0*0.12-0*(-0.18)-0*(-0.14)-1*(-0.16)-1*(-0.11)- 0*(-0.11)-0*(-0.076)-0*(-0.25)-0 *(-0.27)- 0*(-0.25)- 0*(-0.21)= -3.31<br>σ=0.54; t=12<br>12-year disability- and dementia-free survival= exp(-(exp(-3.31) * 12)$^{(1/0.54)}$=0.806<br>Healthy longevity index= disability- and dementia-free survival*100=80.6
